# A unified therapeutic theory for treating cancer via master regulators of the universal apoptosis network

**DOI:** 10.1038/s41420-026-03066-2

**Published:** 2026-04-01

**Authors:** Davis Joseph, Florian Kongoli, Fukka You, Haruhiko Inufusa

**Affiliations:** 1 Division of Anti-Oxidant Research, Life Science Research Center, Gifu University, Gifu, Japan; 2 Flogen Technologies Inc., Mount Royal, QC Canada; 3 FLOGEN Technologies inc., Wilmington, DE USA

**Keywords:** RNA-binding proteins, Apoptosis

## Abstract

Three universal types of cancer are identified, based on the malfunction of a certain set of proteins and regulatory RNAs, irrespective of the organ in which they are located: Cancer Type 1, where cancer cells lack either a functional (a) P14ARF gene, or (b) a P53 gene; Cancer Type 2, where cancer cells lack a functional DINO lncRNA; and Cancer Type 3, where cancer cells have abnormally high MDM2 protein activity. New therapeutic targets were discovered for each type of cancer that pave the way for treating cancer irrespective of the organ it lies in. Until now, current cancer treatments have been organ-specific, and no common pan-organ denominator has been identified. Furthermore, cancer biochemistry has been studied in isolation, one pathway at a time, without considering the complex interactions between proteins and regulatory RNAs, which are characteristic of a living human organism. This work develops a new unified therapeutic theory that identifies novel master regulators of apoptosis as targets for treating cancer regardless of which organ the cancer lies in, through a novel biochemical flowsheet of a universal apoptosis network comprising approximately 100 pathways (80% activation and 20% inhibition), based on a critical analysis of 172 scientific publications that considered all the complex interactions between proteins and regulatory RNAs.

## Facts

1. As of today, there have been many cancer-related articles in the literature dealing with isolated proteins and pathways that have been studied separately and in isolation. This paper uses information derived from a critical review of all the literature as the basis for creating a universal, unique, and unified theory of cell death pathways.
2. As of today, cancer clinical studies in the literature have been mainly organ-specific, and the related basic research articles have studied proteins and their pathways in isolation, without using the proteins to classify cancers. This study uses all available literature data, after critical analysis, to create a unique classification of cancer, based on several exclusive groups of protein function, independently of the organ.
3. As of today, the primary targeting of the HuR protein for cancer treatment relies on the inhibition of HuR activity. This paper, based on critically analyzed literature data introduces a totally new concept of treating cancer via HuR, not by inhibiting HuR itself but by inducing its ability to divide and cause cell death.
4. As of today, creating a method for treating cancer cells with functional P53 genes has been a challenge for the medical community, because no clear molecular target was known. This paper, based on critically analyzed literature data, proposes two new methods of treating cancer cells with functional P53 genes depending on the cause of cancer formation: (1) targeting the DINO lncRNA promoter in DINO-deficient cancer cells and (2) targeting the mir-125b microRNA in cancer with abnormally high MDM2 protein activity.

## Introduction

Cancer, as described in Hajdu et al. [1] was first described in ancient Egypt in 1600 BC, in the Edwin Smith Papyrus. It has since been defined as a group of diseases with abnormal cell growth in different organs. According to Kandoth et al. [2], there are more than 100 types of cancers identified in the literature. Until now, cancer and the fight against it have been characterized by the following three aspects:

1. The classification of cancer has been organ-specific. As a consequence, oncology research has been focused on where the cancer is found instead of what the cancer actually is. As a result, as per André et al. [3] cancer treatment over the last 200 years has been focused on cancers in a specific organ instead of focusing on the nature of cancer at the molecular level, and this has been harmful to patients.
2. Cancer therapy has been an umbrella treatment, with the most usual method being chemotherapy, which often does more harm than good since it damages the healthy cells along with cancer cells. Furthermore, as reported by Mitchell et al. [4], chemotherapy can increase the risk of lung, bladder, colon, and blood cancer, and can also be toxic to many organs, such as the kidney, the heart, the brain, the gastrointestinal tract, the peripheral nervous system, and the genitals; The chances that patients die because of chemotherapy can be equal or higher to the risk of dying from cancer.
3. The cancer biochemical research in recent years has been carried out, and is still currently being carried out in isolation, studying one pathway at a time without considering the complex interactions between proteins and regulatory RNAs, which are characteristic of a living human organism.

The goals of this paper are multifold:

1. Develop a new unified therapeutic theory that identifies novel master regulators of apoptosis as targets for treating cancer regardless of which organ the cancer lies in.
2. Develop a biochemical flowsheet of the universal apoptosis network based on a critical analysis of the scientific publications that considers all the complex interactions between proteins and regulatory RNAs.
3. Develop a method of cancer classification based on the malfunction of a certain set of proteins and regulatory RNAs, irrespective of the organs in which they are located.
4. Develop a new unified therapeutic theory that identifies novel master regulators of apoptosis as targets for treating cancer regardless of which organ the cancer lies in.
5. Discover new universal and specific therapeutic targets for the types of cancers as per the new classification mentioned above, and direct biological antioxidants against cancer cells to induce their deaths.

This paper focuses on two proteins and one microRNA involved in oxidative stress and how they can be targeted in tumors to induce cancer-specific cell death: (1) HuR, (2) P53 and (3) Mir-125b.

This is because, among others, as reported by Kim et al. [5], HuR promotes the production of antioxidant proteins such as SOD2. As described in Liu et al. [6], P53 increases the production of antioxidants proteins such as sestrin and glutathione peroxidase, but induces the death of the cell under apoptotic conditions, if DNA damage is too high. Mir-125b, as described by Pelullo et al. [7], provides protection to the cell against cystic fibrosis-induced oxidative stress.

## The three biological nodes of cancer: HuR, P53 and Mir-125b

As explained in Hinman et al. [8], the Hu antigen R protein (HuR) is widely expressed in eukaryotic cells, which have a nucleus containing DNA. Gorospe [9] describes that HuR is involved in gene expression regulation. Gene expression is the process of protein production based on the instructions provided by the nucleic acid bases in genes encoded in DNA. For instance, after DNA damage caused by UV light, Wang et al. [10] demonstrated that HuR expression was increased in the cytoplasm, the part of the cell outside the nucleus. HuR subsequently bound to p21 messenger RNA (mRNA), stabilizing it. This stabilized messenger RNA leads to increased production of the p21 protein, which is a protein that, as described by Deng et al. [11] prevents the replication of cells with damaged DNA. This prevention, as per Deng et al., is conducted at the G1 phase of the cell’s life cycle, a process known as cell cycle arrest. Qi et al. [12] showed that cell cycle arrest represses apoptosis, also known as cell death. As seen in Bertoli et al. [13], the G1 phase is the period of the cell’s life where proteins known as cyclin-dependent kinases (CDKs) cause DNA replication, which promotes cell division.

HuR also plays an important role in apoptosis, in promoting both proapoptotic and antiapoptotic activity, depending on the amount of damage the cell has undergone. For instance, as discovered by Lal et al. [14], HuR was found to bind with prothymosin α (ProT α) mRNA and stabilize it after cells are exposed to ultraviolet light damage. As seen in Fan et al. [15], HuR binds to adenosine and adenylate/uridylate-rich elements (AREs) of the mRNA in the 3’ untranslated region in the nucleus. This protein-mRNA complex exits the nucleus to the cytoplasm, and HuR protects the mRNA from degradation during and after the exit from the nucleus. This results in the accumulation and increased translation of ProT α. This is important because, as per Jiang et al. [16], ProT α prevents apoptosis by inhibiting the apoptosome’s activity. Abdelmohsen et al. [17] state that HuR stabilizes the mRNA of the SIRT1 protein and increases its production. Only when the cell undergoes oxidative stress did Abdelmohsen et al. find that hydrogen peroxide (H_2_O_2_) activates the cell cycle checkpoint kinase 2 (Chk2) which phosphorylates HuR at S100, leading to dissociation between HuR and SIRT1 mRNA and a subsequent decrease in SIRT1 production. Vaziri et al. [18] demonstrated that SIRT1 removes an acetyl molecular group from lysine 382 in P53 and this reduces its ability to activate transcription. Luo et al. [19] demonstrated that SIRT1’s yeast homolog, Sir2ɑ, was able to promote cell survival by inhibiting P53’s transcriptional activity through deacetylation. Abdelmohsen et al. [20] hypothesized that this was because P53 transcriptional activation function inhibition caused a decrease in the expression of the Bax protein, given that Miyashita et al. [21] reported that P53 is a transcriptional activator of Bax and that Fletcher et al. [22] reported that Bax triggers apoptosis if it is not restrained by pro-survival proteins such as Bcl-2. However, this hypothesis has been challenged by Aubrey et al. [23], who concluded that apoptosis does not require P53-mediated transcriptional induction of the Bax protein. This is based on results from Rathmell et al. [24], who demonstrated that hematopoietic cells expressed normal levels of Bax protein and underwent cell death despite a lack of P53 protein. However, Cohen et al. [25] reported that deacetylases like SIRT1 increased the binding of the Ku70 protein to Bax, and this suppresses the apoptotic activity of Bax.

Another mechanism of apoptosis induction is thus necessary for P53 to lead to cell death. Oda et al. [26] found that P53 caused an increase in the production of Noxa, a Bcl-2 homology 3-only (BH3-only) protein in response to cell damage. Bcl-2 homology 3-only (BH3-only) proteins are proteins which, as described in Huang et al. [27] initiate apoptosis by inactivating Bcl-2 family proteins so that the Bak and Bax proteins can initiate cell death and by directly activating Bak and Bax themselves. As described in Antignani et al. [28], Bak and Bax proteins initiate cell death by destabilizing the outer mitochondrial membrane. Czabotar et al. [29] explain that, in order to destabilize the mitochondrial membrane and kill the cell, identical subunits (monomers) of Bak and Bax must bind together in a process called oligomerization in order to form pores in the mitochondrial membrane. Dewson et al. [30] gives the following thorough description of the nucleation step which initiates this process for the Bak protein: two identical subunits of Bak must first bind together in order for the pore to form, and this binding is caused by the BH3 domain, found in the ɑ2 helix of one Bak protein, which is exposed for binding to ɑ3, ɑ4 and ɑ5 of another Bak protein, a region known as the BH3 binding group. Dewson et al. postulated that BH3 exposure for homodimerization (two identical subunits binding together) is possible because of the BH3 domain in ɑ2 is linked to the ɑ1 helix by a disordered loop, described by Moldoveanu et al. [31] who reported a crystal structure of a Bak homodimer. Subsequent oligomerization of Bak following homodimerization nucleation was found by Dewson et al. [32] to be caused by crosslinking between cysteine residues located in the ɑ6 helices of Bak proteins. Dewson et al. [33] demonstrated that nucleation and subsequent oligomerization of Bax occurs via BH3 domain binding to the groove and subsequent ɑ6 helix cysteine crosslinking, respectively, just like Bak.

Proapoptotic BH3 domain-only proteins have been shown to directly activate Bak and Bax. Desagher et al. [34] reported that the proapoptotic BH3-only protein Bid translocates from the cytosol to the mitochondria, triggers a conformational change in the Bax proteins and leads to the release of cytochrome c. It should be noted that, according to Li et al. [35], Bid only becomes active and translocates to the mitochondria after being cleaved by caspase 8 and transformed into truncated Bid (tBid). As per Beaudouin et al. [36] caspase 8 is activated in the death-inducing signaling complex (DISC). As per Kischker et al. [37] apolipoprotein A1 (APO-1), a tumor necrosis factor receptor, also known as CD95 (or Fas), forms DISC upon association with cytotoxicity-dependent APO-1 associated proteins 1 to 4 (CAP 1 to CAP 4). Jeong et al. [38] explain that the Fas-associated death domain (FADD), which is CAP1 and CAP2, as per Medema et al. [39], acts as a linker between CD95 and procaspase 8 by binding to the death effector domains (DEDs) of the latter. As per Hughes et al. [40], the DISC induces dimerization and self-cleavage of procaspase-8 isoforms 8/a and 8/b to form caspase 8 and initiate cell death. This process, discovered by Scaffidi et al. [41], has been summarized as follows by Lavrik et al. [42]: the 55 kilodalton (kDa) isoform procaspase 8/a and the 53 kDa procaspase 8/b are both cleaved at aspartate 374 to produce the p43 and p41 products, respectively, as well as two copies of p12. P43/41 are subsequently cleaved at Asp216 to produce two copies of p18 and the p26 and p24 products. As per Medema et al., p12 is cleaved at Asp384, forming p10. Lavrik et al. show that both copies of p10 and p18 combine to form the active caspase 8 heterotetramer, which cleaves and activates Bid, that induces cell death via Bax and Bak pore formation. Hughes et al. also reported that procaspase 8 can cleave the c-FLIP protein, a protein which, in its cleaved form (I refer to it as tc-FLIP), as per Kataoka et al. [43], and when expressed at high levels, activates the NF-KB protein, a pro-survival protein which increases the production of HuR. As per Han et al. [44], upon activation, caspase 8 is dissociated from FADD.

Eskes et al. [45] reported that Bax binding to Bid triggered Bax oligomerization and subsequent cytochrome release. Cartron et al. [46] demonstrated that Bax activation from both Bid and Puma, another BH3-only protein, was a result of the interaction between the ɑ1 helice of Bax and the BH3 domain of Bid or Puma. This is important, because Carton et al. [47] stated that this helix, located in the N-terminal, is essential for Bax binding to mitochondria, and Nechushtan et al. [48] showed that Bax N-terminal exposure occurs specifically after Bax activation and for mitochondrial binding.

This entire process is dependent on P53, because Sax et al. [49] demonstrated that Bid transcriptional activation is caused by P53.

According to Vogler et al. [50], there are 6 antiapoptotic BCL-2 family proteins: BCL-2, BCL-XL, BCL-w, MCL-1, BCL2A1, and BCLB. Ishimaru et al. [51] confirmed that BCL-2 production is increased by HuR in cancer cells and Chiou et al. [52] also showed that HuR increases MCL-1 production in leukemia cells. Ishimaru et al. [51] also demonstrated that HuR increases the stability of BCL-2 mRNA, preventing its degradation and thus increasing BCL-2 production. Antiapoptotic BCL-2 family proteins are physically distinguishable from their proapoptotic counterparts, because according to Lomonosova et al. [53], they have four conserved homology domains, BH1 to BH4, and their proapoptotic counterparts, Bax, Bak and Bok, contain only BH1 to BH3, while other proapoptotic members contain only BH3.

Although proteins that conserve BH1-4 function in preventing apoptosis, they have distinct roles. For instance, Fiebig et al. [54] showed that BCL-2 found in the endoplasmic reticulum (ER), where secretory and membrane proteins are produced, could inhibit apoptosis caused by molecules such as ceramide and thapsigargin, but not apoptosis caused by doxorubicin or tumor necrosis factor ɑ (TNFɑ). Also, etoposide-induced apoptosis was prevented by BCL-Xl in fibroblasts, but not by BCL-2. According to Fiebig et al., BCL-XL was also 10 times more effective than BCL-2 at repressing doxorubicin-induced cell death in Michigan Cancer Foundation-7 (MCF-7) breast cancer cells. The method used in Fiebig et al. for measuring the rate of apoptosis is the cleavage of poly (ADP-ribose) polymerase (PARP), because, according to Oliver et al. [55], PARP is extremely sensitive to cleavage caused by apoptosis.

Fletcher et al. [22] showed that binding of pro-survival BCL-2 proteins such as BCL-XL to Bax was necessary to prevent apoptosis. Billen et al. [56] also showed that BCL-XL inhibits the apoptotic activity of tBid by interacting with tBid and preventing its binding to Bax, and Leitl et al. [57] reported that BCL-XL also inhibits the proapoptotic Bak protein. Ku et al. [58] demonstrated that Bax and Bak are inhibited by BCL-2 when the latter binds to the BH3 domain of either proapoptotic proteins. Reynolds et al. [59] also demonstrated that the MCL-1 protein could delay apoptosis caused by c-Myc overexpression. This is similar to what was observed in Talwar et al. [60], who showed that the cleaved product 1 of HuR inhibited the overproduction of c-myc. Apoptosis caused by increased c-myc production was first discovered by Askew et al. [61]. in the murine interleukin 3-dependent myeloid cell line 32D and confirmed by Evan et al. [62]. in fibroblast cells. Askew et al. showed that under conditions where cells are deprived of interleukin 3, a protein which, as per Brizzi et al. [63], causes cell proliferation, cells cease to produce c-myc and ornithine decarboxylase (ODC). Miller et al. [64] describe c-myc as a protein which drives rapid cell growth and division and Wu et al. [65] explain that ODC is necessary for the synthesis of polyamines, a product necessary for cell growth. Therefore, we postulate that c-myc and ODC production decreases in cells deprived of IL-3 to prevent uncontrolled cell proliferation and to stop cell division. However, Askew et al. reported that cells which persisted in producing an increased amount of c-myc even when deprived of IL-3 also increased ODC production. However, instead of proliferating, the increased amount of c-myc in these cells, which were supposed to stop growing, caused cell death. The mechanism by which increased c-myc expression produces cell death is described as follows: As seen in Zindy et al. [66]. an increase of c-myc production increases P19ARF gene expression in mice (P14ARF in humans), a gene which, as described by Zindy et al., responds to signals which can induce hyperproliferation. P53 overproduction occurs after increased P19ARF production as a response to myc overproduction, leading to apoptosis. Zindy et al. found that cells which lacked functional P53 or P19ARF became immortal in response to c-myc overexpression. This was confirmed by Eischen et al. [67]. P19ARF, described as a tumour suppressor by Kamijo et al. [68], was reported in Kamijo et al. [69] to interact with P53 and the mouse double minute 2 homolog (MDM2) protein and increase P53-dependent gene transcription activation. MDM2 is, as per Momand et al. [70]. an inhibitor of P53 activity. Pomerantz et al. [71] determined that the P19ARF-MDM2 interaction prevented MDM2 from inhibiting P53. Ghosh et al. [72] showed that HuR was found to increase the translation of MDM2, demonstrating yet another mechanism for HuR to inhibit apoptosis. Amir et al. [73] reported that Mir-125b suppresses the expression of P14ARF and thus prevents the suppression of MDM2-induced inhibition of P53 apoptosis.

These pro-survival proteins therefore inhibit mitochondrial membrane pore-forming proteins and prevent cell death.

However, these pro-survival proteins are deactivated by BH3-only proteins. Kale et al. [74]. Classify BH3-only proteins into two categories: activator BH3-only proteins (BID and BIM), which bind directly to Bak and Bax, and sensitizer BH3-only proteins (BAD and NOXA), which indirectly promote apoptosis by inhibiting antiapoptotic BCL-2 family proteins.

Yu et al. [75] showed that the Puma protein caused rapid apoptosis in colorectal cancer cells, because it contained a BH3-only domain which inhibits BCL-2 and BCL-XL, consistent with sensitizer BH3-only protein activity. Nakano et al. [76] reported that PUMA production is caused by P53.

Chen et al. [77] reported that NOXA triggered apoptosis by binding specifically MCL-1 and A1 (BFL-1), whereas the BAD protein triggered apoptosis by binding tightly to BCL-2, BCL-XL and BCL-W. Werner et al. [78] confirmed that BFL-1 inhibits apoptosis by sequestering tBID, preventing the activation of Bak and/or Bax. Sharma et al. [79] demonstrated that P53 kills cancer cells in the same way as the cisplatin chemotherapy drug by inducing the production of NOXA. Jiang et al. [80] also reported that BAD gene transcription is activated by p53.

In summary, we conclude that HuR inhibits the entire p53-mediated apoptosis network through its increased production of the SIRT1 protein.

As per Brunet et al. [81], SIRT1 and FOXO3 formed a complex in response to oxidative stress, which prevented FOXO3 from inducing cell death.

According to Brunet et al. [82] the FOXO3 protein, short for forkhead box O3, as defined in Morris et al. [83], is a transcription factor which, in certain contexts, promotes apoptosis by increasing the transcription of the Fas ligand gene. The apoptosis activity of FOXO3, also known as the forkhead transcription factor like 1 (FKHRL1), is inactivated by phosphorylation from the Akt kinase, which also inhibits apoptosis by phosphorylating BAD and caspase 9. According to Friesen et al. [84], the Fas ligand, also known as the CD95 ligand, activates the CD95 receptor (APO-1), which, as previously discussed in this paper, initiates apoptosis. The findings of Dijkers et al. [85] confirm that FOXO3 also triggers apoptosis by increasing the expression of the protein Bim, a BH3 only protein, and this was also confirmed by Sunters et al. [86] in cancer cells. O’Connor et al. [87], who first discovered the protein, showed that BIM can be found in three isoforms: BimEL (the longest), BimL and BimS (the shortest). O’Connor et al. state that the shortest isoform is the most potent for apoptosis, and that Bim triggered apoptosis by inhibiting BCL-2, BCL-XL and BCL-W. As per Chi et al. [88], Bim activates the Bax protein and initiates apoptosis thanks to its BH3 domain and its carboxyl-terminal sequence (CTS), ranging from P121-H140, which binds to the mitochondria and directly interacts with Bax. If CTS was deleted, BIM could still trigger apoptosis, but only by inhibiting BCL-XL. As per Sarosiek et al. [89], Bim has a higher affinity for Bax than for Bak, whereas Bid binds preferably to Bak. Yang et al. [90] showed that Bim caused doxorubicin induced apoptosis by inhibiting the antiapoptotic function of BCL-XL. As per Han et al. [91], though P53 does not directly increase the production of BIM, it does derepress Bim from the inhibitory effects of proteins such as MCL-1 by increasing the production of NOXA, thereby increasing the activity of BIM.

However, Gomez-Bougie et al. [92] discovered that the antiapoptotic protein MCL-1 sequesters and prevents Bim from activating Bax and initiating apoptosis, because, as per Czabotar et al. [93], Bim lacks a c-terminal sequence found in the BH3 domain of NOXA which allows the latter to initiate the degradation of MCL-1.

As described in Hagenbuchner et al. [94], MCL-1 lacks the BH4 domain commonly found in antiapoptotic BCL-2 proteins. In its place, MCL-1 contains proline, glutamic acid, serine and threonine-rich sequences (PEST). As per Domina et al. [95], this sequence, when phosphorylated at the Threonine 163 site by ERK, stabilizes MCL-1 and prevents it from degrading. Germain et al. [96] and Willis et al. [97] confirmed that MCL-1 also inhibits apoptosis by inhibiting BAX and BAK directly.

Brunet et al. explain that SIRT1 deacetylation reorients FOXO3 from increasing the transcription of proapoptotic proteins to increasing the expression of stress response proteins such as GADD45 which, as per Tran et al. [98], stimulates the DNA repair pathway. Chandramouly [99] describes that the repair pathway orchestrated via GADD45 is the nucleotide excision repair pathway.

As per Singh et al. [100], the HuR protein increases the production of GrB10, a protein which activates Akt, and this inhibits apoptosis. Also, Akt activates NF-KB, which increases the production of HuR, a concept known as a positive feedback loop.

Paradoxically however, Mazan Mamczarz et al. [101] found that HuR actually enhances p53 expression in cells in response to UV light damage, and Ahuja et al. [102] reported that HuR stops Mir-125b from repressing P53 activity, creating a pulse-like production of P53, as reported by Guha et al [103]. and Goswami et al. [104]

Donahue et al. [105] also demonstrated that increased production of the HuR protein increased the expression of the P53 protein, a protein which, as seen in Levine et al. [106], suppresses cancer progression. As seen in Adamkov et al. [107], increased p53 protein production inhibits the expression of the survivin protein, a protein which, as described in Donahue et al., inhibits apoptosis. Hoffman et al. [108] demonstrated that p53 represses survivin production at the transcriptional level, by binding to the survivin gene promoter in vivo. Therefore, HuR inhibits survivin overexpression by increasing p53 levels, thereby reducing the protein levels of this antiapoptotic protein. However, as described in Wasylichen et al. [109], most cancer cells inhibit P53 activity. If p53 is not expressed, Donuahue et al. observed that HuR increases the production of survivin instead of decreasing it. Thus, HuR increases the production of the proapoptotic surviving protein in cancer cells but represses it in healthy cells.

Under certain circumstances, however, HuR can promote apoptosis rather than prevent it when cell damage is severe and beyond repair. HuR, like its paralogs, has 3 RNA recognition motifs (RRMs). As seen in Ma et al. [110] the first two RRMs bind to AREs and the third motif binds to the polyadenylated tail. Fan et al. also proved that HuR is able to shuttle between the nucleus and the cytoplasm because of a nuclear cytoplasmic shuttling sequence (HNS) located between the second and a third RRM. Brennan et al. [111] demonstrated that HuR binds to four different proteins: (1) the suppressor of variegation 3-9 (Su(var)3-9), Enhancer of zeste (E(z)), and Trithorax (Trx) α, SET α for short (protein name found in Herz et al. [112], (2) SET β, (3) phosphoprotein 32, pp32 for short, and (4) acidic protein rich in leucine, APRIL for short. Brennan et al. showed that HuR binds to these four proteins via RRM3 and HNS. Brennan et al. determined that pp32 and APRIL, like HuR, were able to move from the nucleus to the cytoplasm and vice-versa using a heterokaryon assay described in Schmidt-Zachmann et al. [113], where cells from different species are fused and the ability of a protein to shuttle from the nucleus to the cytoplasm is determined by detecting the presence of a protein from one species (tagged with immunofluorescence) in the nucleus of the other species of cell, which was the case for both pp32 and APRIL. Brennan et al. also determined that both pp32 and APRIL contain leucin-rich nuclear export signals (NES) necessary for binding to the chromosomal maintenance 1 protein, also known as CRM1 or exportin 1. Fornerod et al. [114] found that RanGTP binding to CRM1 allowed CRM1 to bind to the cargo protein which was to be exported outside of the nucleus, a process known as cooperative binding. Melchior et al. [115] identified Ran GTP as an essential transport factor between the nucleus and the cytoplasm. Neville et al. [116] discovered that CRM1 acts as an intermediary between the cargo protein it carries and the nuclear pore complex. Neville et al. showed that CRM1 interacts with the receptor-interacting protein 1 protein (Rip1p), which, as per Stutz et al. [117], functions as a docking protein for the nuclear pore to unload proteins outside of the nucleus. As per Waldmann et al. [118], the human ortholog of Rip1p is the nucleoporin-like protein NLP1.

Pp32 is of particular interest for this study because, as seen in Chakravarti et al. [119], pp32, also known as PHAP1, stimulates apoptosis, and Mazroui et al. [120] demonstrated that, when the cell undergoes lethal damage, HuR exits the nucleus along with pp32. When it reaches the cytoplasm, HuR is cleaved by caspases 3 and 7 at aspartate 226 and this stimulates apoptosis. Von Roretz et al. [121] determined that the HuR cleavage products, cleavage product 1 and cleavage product 2 (CP1 and CP2), selectively bind to procaspase 9 mRNA during the early stages of apoptosis, increasing its stability and caspase 9 protein production. However, they erroneously refer to procaspase 9 mRNA as caspase 9 mRNA. This is a mistake, there is no such thing as caspase 9 mRNA, because, as seen in Wu et al. [122] caspase 9 is a dimer of two procaspase 9 proteins. This is important, because, as per Fadeel et al. [123], caspase 9 (the activated form of procaspase 9) forms the apoptosomes, along with cytochrome c and the apoptotic protease-activating factor 1 (apaf-1) as seen in Zhou et al. [124] which activates other caspases such as caspase 3, a proteolytic enzyme that degrades proteins and leads to cell death.

Von Roretz et al. [125] proved that HuR-CP1 advances apoptosis by binding transportin 2 and preventing it from re-importing HuR to the nucleus. This paper however misinterpreted HuR-CP2’s role in promoting apoptosis. It claimed that HuR-CP2 triggers apoptosis by binding to PHAP1 (pp32) when it fact, it was later proven in a subsequent paper with the same first author that both HuR-CPs trigger apoptosis by binding to caspase 9 mRNA and increasing its translation as seen above. Rebane et al. [126] demonstrated that transportins 1 and 2 are involved in the import of HuR into the nucleus. This finding was confirmed specifically for transportin 2 by Güttinger et al. [127], who described transportin 2 as an importin of HuR to the nucleus. Giessen et al. [128] also demonstrated that preventing HuR import by disrupting the HuR-transportin 2 complex was a key step in muscle tissue development.

**Fig. 1:**
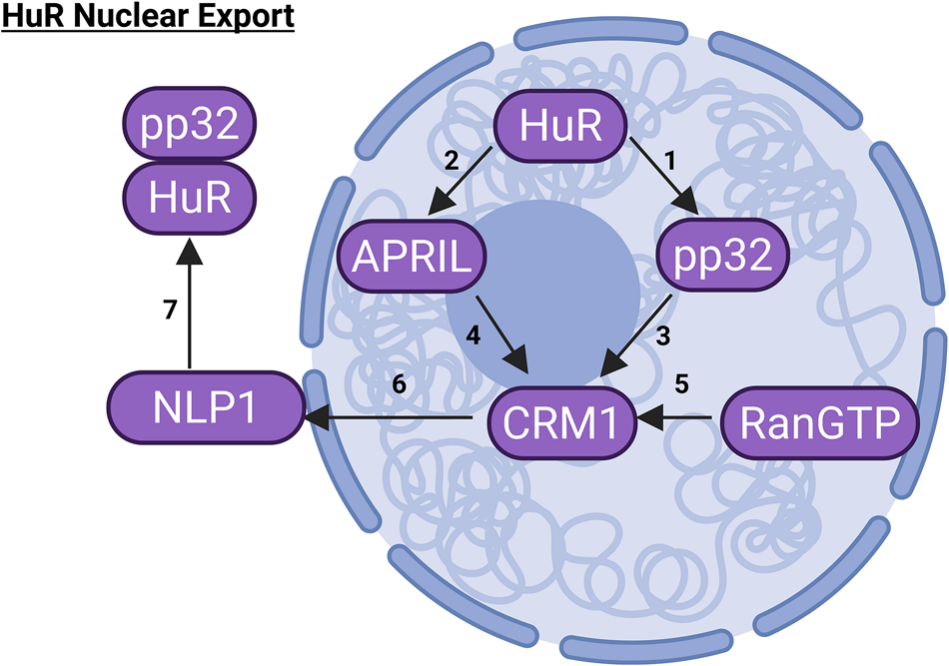
A comprehensive new biochemical flowsheet of the mechanistic pathway for the shuttling of the Human antigen R (HuR) protein outside of the nucleus.

**Fig. 2:**
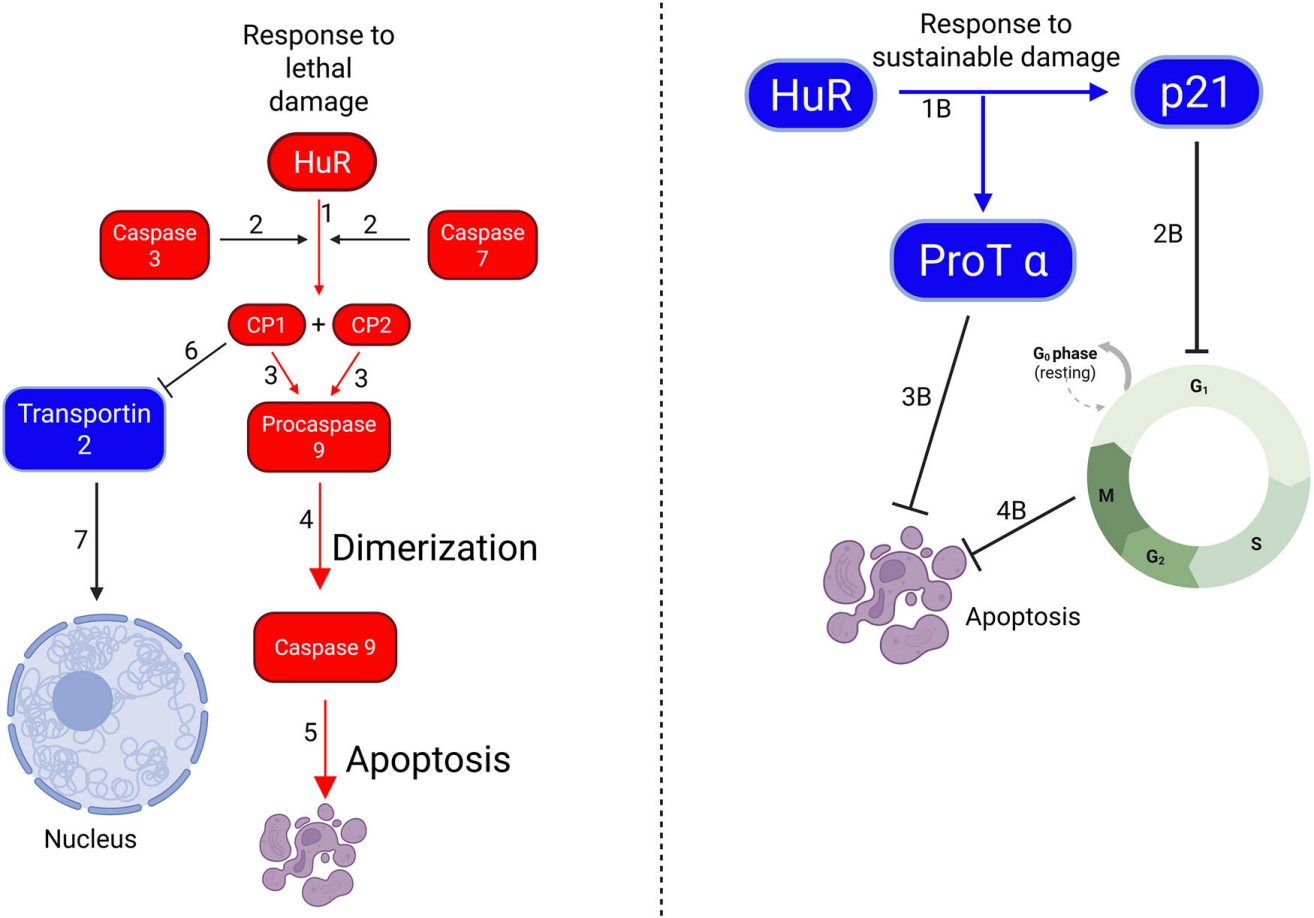
A new comparative biochemical flowsheet analyzing the differences in the response of Human antigen R (HuR) to sustainable and lethal cell damage.

The biochemical flowsheets of HuR shuttling outside the nucleus and regulating apoptosis are shown in Figs. 1 and 2 below (all figures in this manuscript were created using Biorender.com):

Steps 1 and 2 are HuR binding to either the APRIL or pp32 proteins, respectively. Steps 3 and 4 are APRIL and pp32, respectively, binding to CRM1 for shuttling outside of the nucleus. Step 5 is RanGTP binding to CRM1 to enable the shuttling. Step 6 is CRM1 docking on the R1p1p human ortholog NLP1 in order to be ejected into the cytoplasm.

In response to lethal damage, step 1 shows the cleavage of HuR into CP1 and CP2. Step 2 shows the cleavage being performed by caspases 3 and 7. Step 3 shows CP1 and CP2 inducing the overproduction of procaspase 9. Step 4 shows procaspase 9 production triggering dimerization and caspase 9 activation, and step 5 shows apoptosis. Step 6 shows the inhibition of transportin 2, preventing transport of HuR to the nucleus in step 7.

In response to sustainable damage, step 1B shows the production of Prot α and p21. Step 2B shows cell cycle arrest at the G1 phase caused by P21. Step 3B shows Prot α inhibiting apoptosis and step 4B shows cell cycle arrest caused by P21, thus inhibiting apoptosis.

HuR’s involvement in cell death and maintaining mRNA stability has been linked to the development of cancer (oncogenesis). A clear link between HuR and cancer can be found in Fan et al., which demonstrated that HuR is overexpressed in cells with the highest rate of proliferation / division, a hallmark of cancer cells. One such implication of HuR in cancer was seen above regarding HuR’s involvement in the p53 and survivin pathways.

Moreover, Kurosu et al. [129] reported that HuR increases the production of the vascular endothelial growth factor A (VEGF) and cyclooxygenase 2 (COX-2). This is important, because Kim et al. [130] reported that VEGF is a tumour growth factor, and that inhibiting VEGF activity decreased blood vessel density in tumours and suppressed tumour growth. As mentioned in chandrasekharan et al. [131], COX-2 produces prostaglandins, and Xu et al, [132] identified prostaglandin E2 as an angiogenic factor. These factors play a key role in increasing blood vessel density in tumours. As per Durie et al. [133], HuR also increases the production of the XIAP protein, which is, as per Scott et al. [134], an inhibitor of caspases 3, 7 and 9. XIAP has been described as an apoptosis inhibitor by Deveraux et al. [135], with distinct fragments in the protein inhibiting one specific caspase, such as the baculoviral inhibitory repeats 1 and 2 which inhibit caspases 3 and 7, and repeat 3, which inhibits caspase 9. Nabors et al. [136] also reported that HuR increased the expression of other angiogenic factors such as TGF-*β* and interleukin-8 (IL-8). TGF-*β* was reported by Ferrari et al. [137] to cause angiogenesis through apoptosis caused by VEGF signalling (this is a unique context where VEGF is proapoptotic instead of antiapoptotic) and Shi et al. [138] state that interleukin 8 is known to promote angiogenic responses. Folkman [139] reported that angiogenesis inhibitors reduce apoptosis.

Wu et al. [140] and Wei et al. [141] both report on the effectiveness of HuR inhibitors in combatting cancer. Wu et al. identified 1c and 7c as molecules which inhibited HuR by binding to the RNA binding pocket of HuR. Wei et al. describes the KH-3 molecule as a molecular inhibitor of HuR, which, as per Liu et al. [142] disrupts HuR’s interactions with mRNA.

Notably, Wei. et al. explain that HuR increases the production of SLC7A11. This is important, because Dixon et al. [143] showed that SLC7A11 inhibition prevents cystine-glutamate exchange in the cell and this causes ferroptosis. This type of cell death, as described by Dixon et al. [144], is an oxidative form of cell death. Ferroptosis is caused by the reaction between iron and polyunsaturated fatty acids (PUFAs) to form membrane lipid peroxides (lipids containing unstable single-bonded oxygen atoms) that cause cell death. This happens because, as per Dixon et al. [145], the cell is deprived of cystine, which is essential for antioxidant defense. Yan et al. [146] explain that cysteine, derived from cystine, is necessary for producing antioxidant molecules such as glutathione and they also describe that SLC7A11 overexpression can protect cancer cells from oxidative stress. As explained by Lubos et al. [147], glutathione is a cofactor of glutathione peroxidase (GPX), which eliminates hydrogen peroxide, a reactive oxygen species. Zhao et al. [148]. explain that glutathione is also a cofactor involved in the glyoxalase system (GLO 1 and GLO 2), which eliminates oxidative stress-related alpha-carbonyl species. Tang et al. [149] also explain that glutathione is a cofactor of glutathione s-transferase (GST), a crucial antioxidant enzyme. The nucleophilic properties of glutathione catalyze liver detoxification via GST. Jiang et al. [150] found that p53 also causes ferroptosis by inhibiting SLC7A11.

Kataoka et al. [151] reported that an HuR mutant, HuR-V225I, was associated with adult T cell Leukemia/lymphoma. Colalillo et al. [152] later found that this HuR mutant prevents HuR from being exported to the cytoplasm, resulting in increased translation of the pro-apoptotic XIAP and BCL-2 proteins.

HuR’s involvement in cancer is reflected in the abovementioned ways it prevents apoptosis and oxidative stress, and, by doing so, increases cancer proliferation.

Oxidative stress sometimes must be maintained to fight cancer cells, because, as per Chen et al. [153] and An et al. [154], oxidative stress causes autophagy-induced cell death and apoptosis in cancer cells.

The main downside of antioxidants in cancer patients is the removal of the tumor suppressive function of oxidative stress. However, this can be resolved by a combination therapy which (a) maintains the tumor suppressive function of P53, or (b) eliminates the oncogenic effects of HuR.

If the P53 and P14ARF genes remain intact in the tumour, HuR will be able to enhance P53’s activity, only Mir-125b hinders it. Therefore, a combination therapy of antioxidant therapy and Mir-125b inhibitors will allow healthy cells to obtain the beneficial effects of antioxidants while Mir-125b inhibitors prevent mir-125b from hindering p53.

If the P53 gene has been damaged to the point where translation of functional P53 is not a possibility or a harmful, mutated form of P53 is produced, this is where HuR expression becomes problematic. HuR, as seen above, enhances the production of oncogenic proteins such as survivin if functional p53 is absent. Under these circumstances, inhibitors which target the RNA binding pockets of overexpressed HuR in cancer cells can be used.

In summary, the main point of this unified theory is that HuR is an ally in the fight against the tumour if the p53 gene remains intact in the cancer cell and it is Mir-125b, which inihbits HuR functions, that must be targeted for inhibition. However, if P53 can no longer be produced or its harmful mutants are produced instead due to the damage at the genetic level, that is where HuR must be targeted instead for destruction.

Targeting overexpressed HuR in the absence of a functional P53 gene and Mir-125b in the presence of a functional P53 gene in the cancer cell will be the key to repressing tumour growth and proliferation during antioxidant therapy for cancer patients. Marney et al. [155] showed that, in cancer, three modifications (P53 gene impairment, MDM2 amplification and DINO impairment) are mutually exclusive, with DINO suppression being responsible for a significant amount of post-translational P53 impairment in tumours.

The targeting of HuR, Mir-125b or the DINO promoter will depend on the level at which P53 is inhibited. If P53 is inhibited at the genetic level, it is HuR that must be targeted because it will increase survivin production and thus prevent the cell death of the malignant cell, and Mir-125b will play a tumour suppressor role by inhibiting c-myc and preventing it from activating cancerous cell proliferation in P53/P14ARF-deficient cells. If P53 is inhibited at the protein level via MDM2 overproduction, then the activation of the MDM2 suppressor P14ARF must be increased via Mir-125b suppression. If P53 activity is inhibited due to lack of DINO lnc RNA, it is the DINO promoter which must be targeted.

This dichotomy we discovered through our new therapeutic frameworks answers why mir-125b is oncogenic in some cases and acts as a tumour suppressor in others. Shi et al. [156] reported that mir-125b promotes cancer growth in prostate by repressing the production of the bak protein. Mir-125b’s behaviour as a suppressor of p53 at the mRNA level, first demonstrated by Le et al. [157], also allude to mir-125b being an oncogene. However, Nakanishi et al. [158] reported that inhibiting mir-125b in head and neck cancer cells contributed to cancer development, citing a cancerous c myc-activation as a result of Mir-125b inhibition. We postulate that Mir-125b’s repression of P14ARF and P53 may be the cause of cancer resistance to apoptosis in Shi et al. and Mir125b’s inhibition of c-myc may be preventing cancer development in Nakanishi et al. because the P53-P14ARF apoptosis axis may be compromised at the genetic level in the cancer cells studied in this paper.

Marney et al. also confirmed that cancer cells where P53 is intact can survive by silencing the DINO long noncoding RNA (lnc RNA). The silencing is done by hypermethylation at the DINO transcription start site (DMR1). Schmitt et al. [159] first discovered that DINO was required for P53-induced apoptosis.

**Fig. 3:**
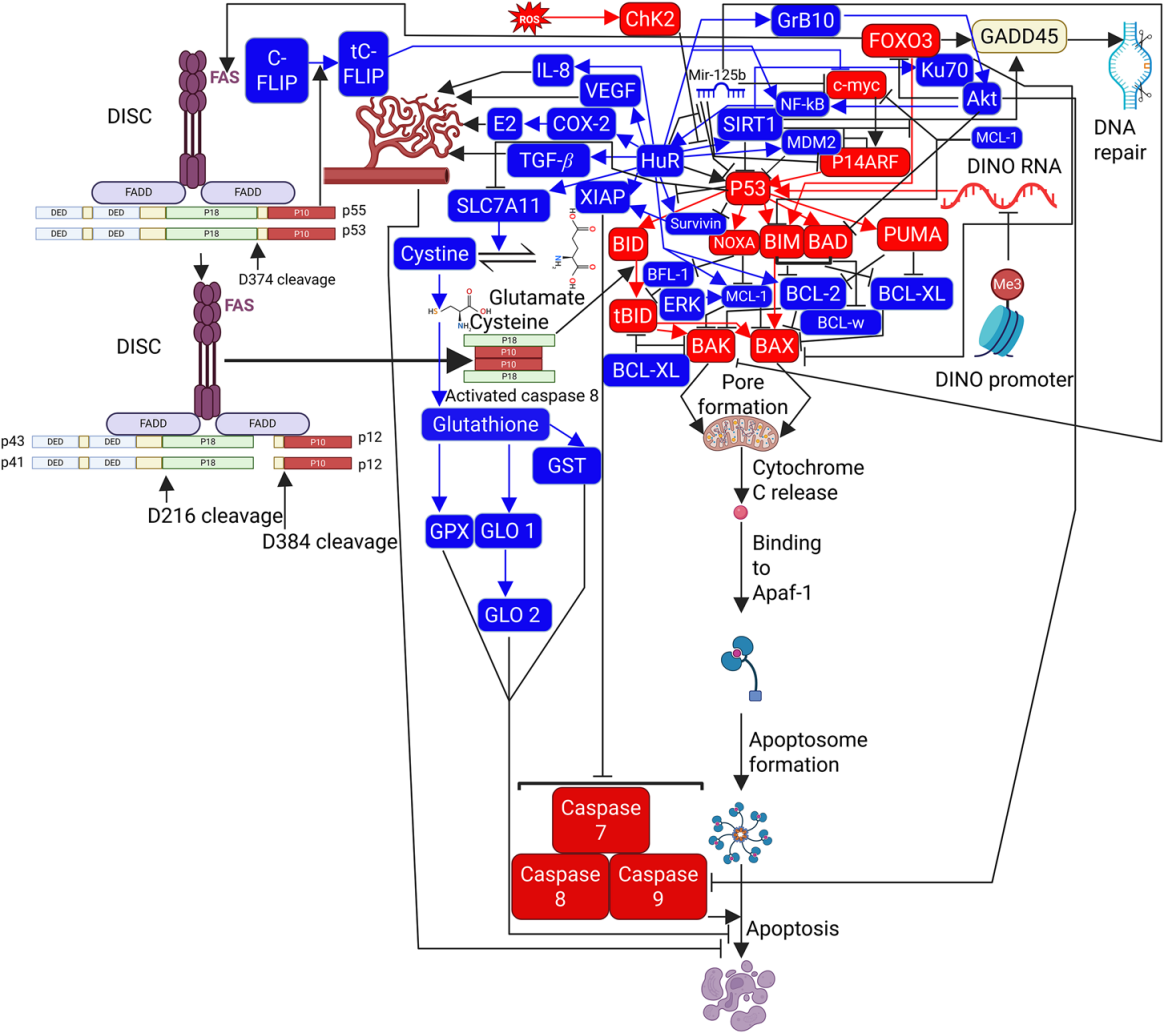
Unified theory on apoptosis (cell death) expressed as a biochemical flowsheet of a universal apoptosis network, with antiapoptotic proteins and signaling shown in blue and proapoptotic proteins and signaling shown in red.

The unified theory of all 100 apoptosis pathways studied in this paper are shown below in Fig. 3.

Altogether, a newly developed definition of apoptosis in the context of the unified theory can be described as follows:

### Mechanistic overview of apoptosis within the universal apoptosis network

Apoptosis is a tightly regulated form of programmed cell death essential for maintaining tissue homeostasis and eliminating damaged or malignant cells. Two principal apoptotic pathways have been described: the extrinsic (death receptor–mediated) pathway and the intrinsic (mitochondrial) pathway. Although historically treated as distinct, extensive cross-talk exists between these pathways, and both converge on a common execution phase mediated by caspase activation.

#### Extrinsic apoptotic pathway

The extrinsic pathway is initiated through the activation of death receptors located on the cell surface, such as Fas (also known as CD95 or APO-1), members of the tumor necrosis factor receptor superfamily. Upon ligand binding, these receptors oligomerize and assemble the death-inducing signaling complex (DISC), which recruits adaptor proteins including Fas-associated death domain (FADD) and procaspase-8. DISC formation induces procaspase-8 dimerization and autocatalytic cleavage, generating active caspase 8.

Activated caspase 8 initiates apoptosis through two mechanisms: (i) direct cleavage and activation of executioner caspases, and (ii) cleavage of the BH3-only protein BID into truncated BID (tBID). tBID translocates to the mitochondria, providing a direct molecular link between the extrinsic and intrinsic apoptotic pathways.

#### Intrinsic (Mitochondrial) apoptotic pathway

The intrinsic pathway is triggered by intracellular stress signals such as excessive DNA damage, oncogene overexpression (e.g., c-MYC), oxidative stress, or metabolic imbalance. A central regulatory role in this pathway is played by the BCL-2 family of proteins, which consists of anti-apoptotic members (e.g., BCL-2, BCL-XL, MCL-1), pro-apoptotic multidomain effector proteins (BAX and BAK), and pro-apoptotic BH3-only proteins (e.g., PUMA, NOXA, BIM, BID).

Upon sufficient apoptotic stress, BH3-only proteins neutralize anti-apoptotic BCL-2 family members and/or directly activate BAX and BAK. Activated BAX and BAK undergo conformational changes, oligomerize in the outer mitochondrial membrane, and form pores in a process known as mitochondrial outer membrane permeabilization (MOMP). This event leads to the release of cytochrome c into the cytoplasm.

Cytochrome c subsequently associates with apoptotic protease-activating factor 1 (APAF-1) and procaspase-9 to form the apoptosome, resulting in caspase-9 activation. Caspase-9 then activates downstream executioner caspases, principally caspases-3 and -7, culminating in the proteolytic dismantling of the cell.

#### p53 as a central apoptotic integrator

The tumor suppressor protein p53 serves as a central integrator of apoptotic signaling by transcriptionally activating pro-apoptotic genes in response to cellular stress. p53 induces the expression of BH3-only proteins such as PUMA, NOXA, BIM, and BID, thereby promoting mitochondrial apoptosis. Additionally, p53 can influence apoptosis through transcription-independent mechanisms at the mitochondria.

Disruption of p53 function—whether through genetic dysfunction, MDM2-mediated degradation, or impaired transcriptional activation due to long non-coding RNA dysregulation—constitutes a major mechanism by which cancer cells evade apoptosis.

#### Integration of HuR within apoptotic regulation

Human antigen R (HuR) functions as a post-transcriptional regulator that modulates apoptosis in a context-dependent manner. Under conditions of moderate cellular stress, HuR promotes cell survival by stabilizing mRNAs encoding anti-apoptotic proteins and cell cycle regulators, thereby facilitating DNA repair and recovery. Conversely, under conditions of lethal or irreparable damage, HuR undergoes cytoplasmic accumulation and caspase-mediated cleavage.

Cleaved HuR fragments selectively stabilize procaspase-9 mRNA, leading to increased caspase-9 production and apoptosome activation. This switch transforms HuR from a pro-survival factor into a direct amplifier of apoptotic execution, positioning HuR cleavage as a critical molecular decision point between survival and programmed cell death.

#### Unified apoptotic execution in the proposed network

In the unified apoptosis network presented in this work, extrinsic and intrinsic apoptotic pathways are viewed as a coordinated system regulated by master molecular nodes rather than isolated linear cascades. The convergence of p53 signaling, BCL-2 family regulation, caspase activation, and HuR-mediated post-transcriptional control provides a cohesive mechanistic framework explaining how diverse oncogenic alterations ultimately determine apoptotic competence or resistance across cancer types.

##### Unified theory on cancer cell apoptosis

Based on the critical analysis above, we determined that cancer can be put into three different families, regardless of the origin of the cancer or the organ containing the tumour:

1. Cancer Type 1: Cancer cells with a defective c-myc overexpression apoptosis pathway, lacking either (a) a functional P14ARF gene, or (b) a functional P53 gene.
2. Cancer Type 2: Cancer cells lacking a functional DINO lncRNA.
3. Cancer Type 3: Tumours in which P53 activity is suppressed at the protein level via dominant MDM2 activity, irrespective of whether suppression is caused by gene amplification or transcriptional upregulation.

**Fig. 4:**
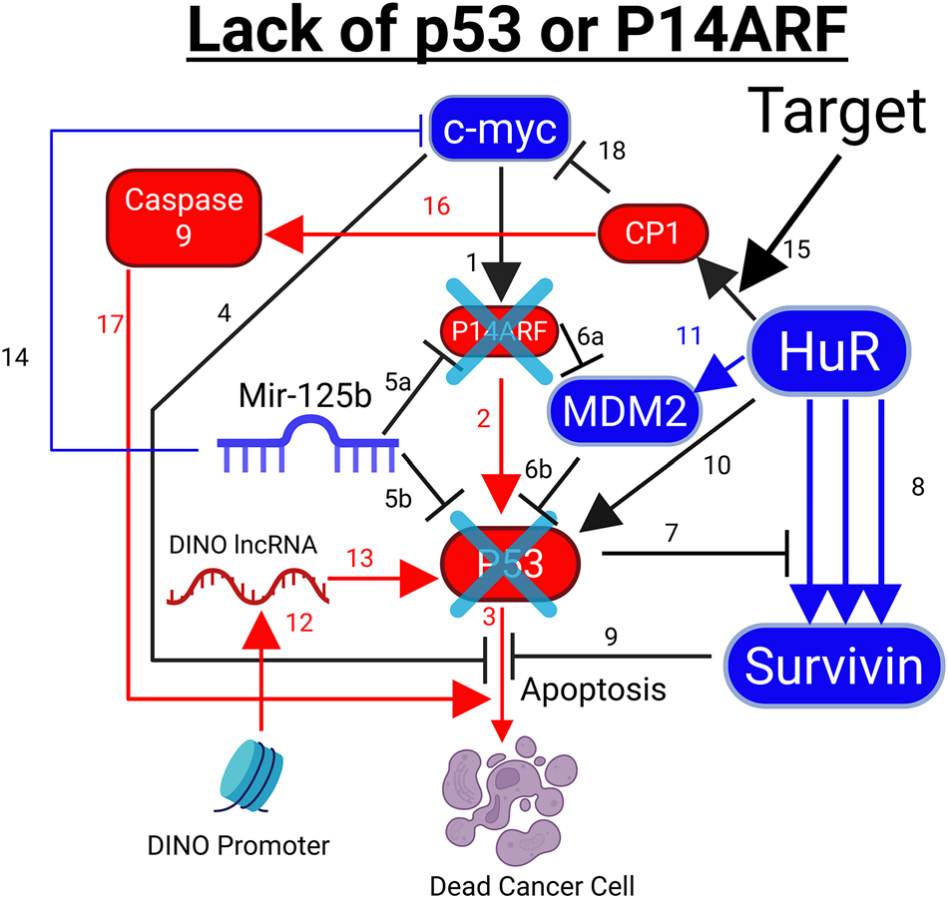
Biochemical flowsheet that unveils for the first time the main therapeutic target for cancer type 1, where either P14 Alternative Reading Frame (P14ARF) or P53 at the genetic level are dysfunctional.

**Fig. 5:**
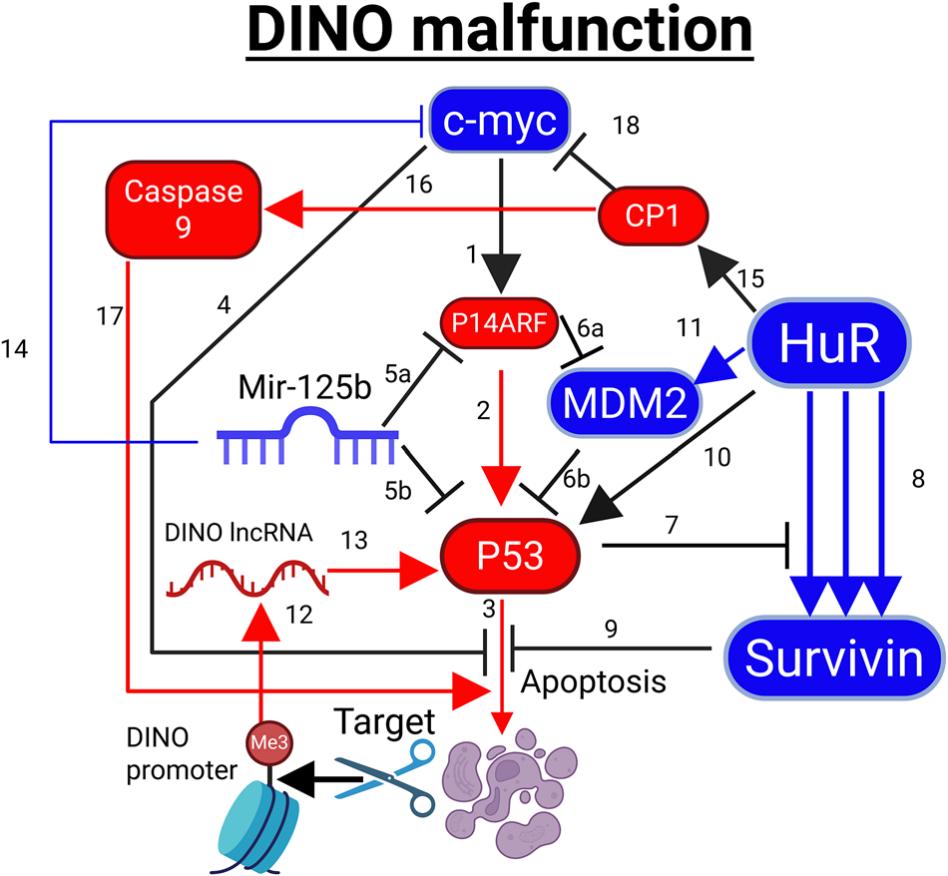
Biochemical flowsheet that unveils for the first time the main therapeutic target for cancer type 2, where Damage Induced noncoding RNA (DINO) long noncoding RNA (lncRNA) production is dysfunctional.

**Fig. 6:**
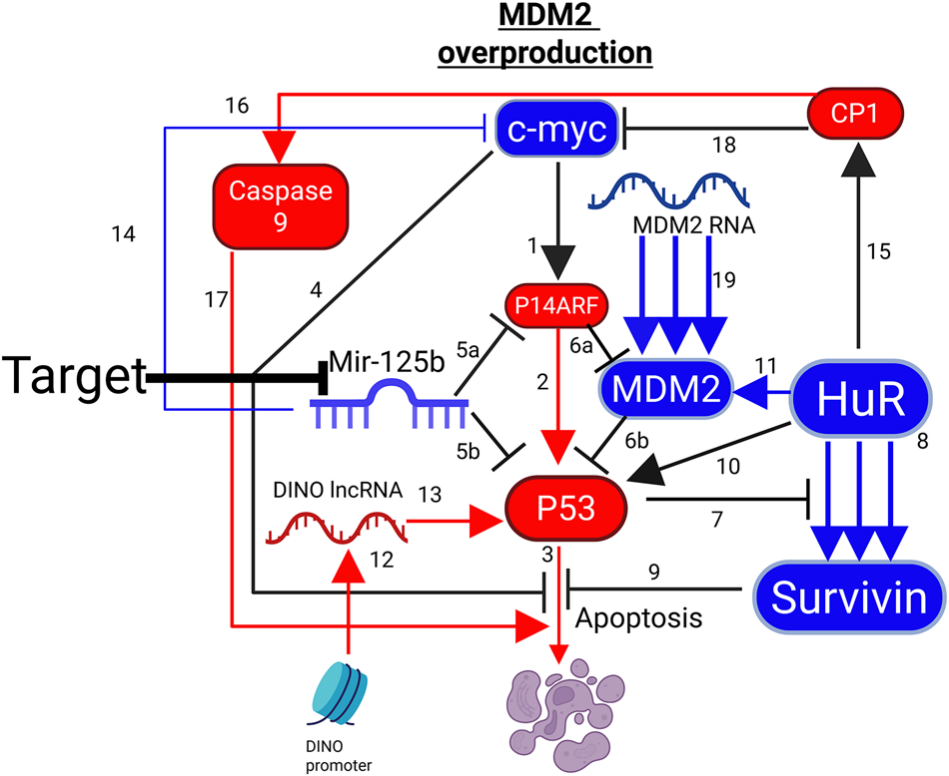
Biochemical flowsheet that unveils for the first time the main therapeutic target for cancer type 3, where (Mouse double minute 2 homolog) MDM2 is overproduced.

All three cancer families can be eliminated using three unique biochemical therapeutic approaches we developed in this paper, shown in Figs. 4–6 below.

**Cancer type 1:** As mentioned above, this proposed cancer type is characterized by cancer cells with a defective c-myc overexpression apoptosis pathway, lacking either (a) a functional P14ARF gene, or (b) a functional P53 gene.

Cancer Type 1 is schematically shown in Fig. 4 below, incorporating and unifying all pathways related to it.

Steps 1 to 3 show the healthy standard apoptosis caused by P14ARF and P53 in response to c-myc overproduction. The X symbols over P14ARF and P53 represent missing functional P53 or P14ARF, which is characteristic of cancer type 1 that we have defined for the first time above.

The end result of losing either protein is the same: c-myc overproduction in the absence of growth factors such as interleukin 3 no longer triggers cell death and, conversely, inhibits apoptosis in step 4, as reported in Zindy et al. This dramatically changes the healthy biochemical interactions in the cell represented by the pathway set 1 to 3. As a result of P53 loss, the inhibitory actions of Mir-125b, P14ARF and MDM2, as shown in 5a, 5 b, 6a and 6 b, respectively, become irrelevant. If P14ARF is lost, it can no longer inhibit MDM2 in step 6a, and MDM2 will become more active and will degrade P53 as seen in step 6b.

Losing P53 function means that this protein can no longer inhibit HuR from increasing the production of the survivin protein, as seen in step 7, and an increased amount of survivin protein further inhibits apoptosis, as seen in steps 8 and 9. HuR, which causes p53 protein production in step 10 and induces MDM2 production for p53 inhibition in step 11, also becomes irrelevant if the p53 gene is defective. If P14ARF is missing, MDM2 production by HuR in step 11 will lead to MDM2 degrading P53 overwhelmingly, because P14ARF will not be present to maintain the proper balance of MDM2 function in step 6a.

In cancer type 1, when P53 is missing at the genetic level, DINO lnc RNA production, as seen in step 12, remains intact because DINO lncRNA malfunction and p53 gene malfunction cannot exist at the same time (mutually exclusive), since the impact of each malfunction is the same (loss of P53 activity), and the malfunction of both simultaneously is therefore not necessary for the survival of the cancer cell, as seen in Marney et al. Because P53 is absent, DINO lncRNA activity that promotes P53 transcription activation, shown in step 13, becomes irrelevant regarding apoptosis.

We postulate for the first time in this paper that DINO lnc RNA malfunction and P14ARF gene malfunction are also mutually exclusive. This is because Kannan et al. [160] reported that inactivation of either p53 or p14ARF alleviates the need for the inactivation of the other. Our postulate is logical, because, as seen in the healthy cell pathway set in steps 1 to 3 in Fig. 4, in the case of c-myc overproduction, a defective P14ARF gene will not allow P53 to trigger apoptosis, because loss of P14ARF increases MDM2 activity and causes P53 degradation. Therefore, because P14ARF gene deletion already renders P53 effectively obsolete in combating cancer, DINO production is not disrupted by the cancer cell.

In the view of the abovementioned analysis, two important conclusions are drawn:

1. Contrary to what is often proposed in the scientific literature, deactivating Mir-125b for cancer treatment would be a bad idea for cancer type 1, because Mir-125b inhibits c-myc overexpression, as seen in step 14 of Fig. 4. Therefore, we propose to avoid Mir-125b for cancer type 1.
2. Contrary to the proposed HuR inhibitors reported by Wu et al. and Wei et al., inhibiting HuR without inducing its cleavage would be a bad idea because it deprives the cell of HuR’s apoptotic functions. Cleaved HuR can no longer induce the production of the antiapoptotic survivin or MDM2 proteins as seen in steps 8 and 11. However, the CP1 fragment (along with CP2), produced by HuR cleavage in step 15, increase the production of caspase 9 seen in step 16, and, as reported by Druskovic et al. [161], overproduction of caspase 9 triggers apoptosis, as seen in step 17. Moreover, the CP1 fragment, as reported by Talwar et al., inhibits c-myc overproduction during hypoxic stress, as seen in step 18. This is important regarding cancer, because, as described by Muz et al. [162], tumours cause hypoxia due to oxygen demand exceeding oxygen supply. These last two abilities of cleaved HuR to induce the death of the cancer cells and prevent tumour growth are lost if you simply inhibit HuR. In view of this analysis, we propose the induction of HuR cleavage, seen in step 15, for cancer type 1 treatment.

In light of our findings, we determined that the therapeutic target for inducing the death of this family of cancer type 1 is the HuR protein cleavage mechanism. In order to properly eliminate P53 or P14ARF-defficient cancer cells, classified in this article as cancer type 1, HuR cleavage initiation is essentially a plan B which directly induces cell death by triggering caspase 9 production when the P53 or P14ARF genes are malfunctioning.

This proposed treatment is likely to overwhelmingly affect cancer cells and not healthy cells because, as reported in Levidou et al. [163], HuR is found in the cytoplasm of malignant cells at significantly higher rates compared to healthy cells. We deduced that a hypothetical drug inducing HuR cleavage will disproportionately target cancer cells, overshuttling HuR in the cytoplasm.

If the cancer cell has developed a HuR mutant to protect itself from apoptotic stress by producing antiapoptotic proteins within the nucleus, the target shifts from cleavage induction to correcting the mutation at amino acid 225 in HuR. Since the cell is already under apoptotic stress in the Colalillo et al. scenario, HuR shuttling out of the nucleus in this specific scenario will allow cleavage to occur naturally.

**Cancer type 2:** As mentioned above, this proposed cancer type is characterized by Cancer cells lacking a functional DINO lncRNA.

Cancer Type 2 is schematically shown in Fig. 5 below, incorporating and unifying all pathways related to it.

The second cancer family involving DINO malfunction has the same pathways as the first. However, the problem lies in the DINO promoter, which is methylated, as reported in Marney et al. Steps 12, 13 and 3 are therefore compromised due to P53 transcriptional activation malfunction, which causes direct apoptosis inhibition, and c-myc induced apoptosis inhibition.

The therapeutic target we propose is the demethylation of the DINO promoter, which will result in proper apoptotic function.

HuR cleavage initiation also works as a plan B for cancer type 2.

**Cancer type 3:** As mentioned above, this proposed cancer type is characterized by Cancer cells with overproduced MDM2.

Cancer Type 3 is schematically shown in Fig. 6 below, incorporating and unifying all pathways related to it.

In cancer type 3, MDM2 is overproduced either due to gene amplification specifically, as reported in Hou et al. [164], or transcriptional upregulation (MCF-7), as seen in step 19, resulting in the degradation of P53 function and the subsequent apoptosis suppression, similar to cancer types 1 and 2. However, unlike cancer type 1, the target cannot be HuR in cancer type 3 if the goal is to kill the cancer cell via P53. The suppression of P53 activity is at the protein level in cancer type 3, caused by MDM2 protein degradation in step 6b. P53 will remain suppressed if HuR is deleted, because HuR is necessary for P53 production as seen in step 10.

DINO treatment is irrelevant in cancer type 3, unlike cancer type 2, because DINO lncRNA production remains intact.

Therefore, we concluded that the target for eliminating cancer type 3 via P53 must be Mir-125b. Mir-125b is a suppressor of both P53 and P14ARF, as seen in steps 5a and 5b of Fig. 6. Eliminating Mir-125b will increase P53 activity. More importantly, the elimination of Mir-125b will increase the activity of P14ARF. This is important because P14ARF is an inhibitor of MDM2. Eliminating Mir-125b in this cancer type would increase the inhibition of MDM2 by P14ARF while simultaneously increasing P53 activity directly and indirectly.

HuR cleavage initiation also works as a plan B for cancer type 3 in a p53-independent manner.

We propose that MCF-7 breast cancer cells exhibit a protein-level p53 suppression phenotype consistent with Cancer Type 3, despite retaining a wild-type TP53 gene. Apoptosis is suppressed in these cells in part because of impaired apoptotic execution due to caspase 3 deficiency, as seen in Essman et al. [165]. Multiple studies, such as Zhou et al. [166] report elevated MDM2 expression in MCF-7 cells. We postulate that this is a significant contributing factor to functional restraint of p53 activity in these cells, and Portman et al. [167] further showed that MDM2 inhibition restores apoptotic signaling in MCF-7 cells. Therefore, MCF-7 cells demonstrate the defining biochemical feature of Cancer Type 3: suppression of p53 function at the protein level through dominant MDM2 activity. Furthermore, induction of HuR cleavage for cancer type 3 may provide a tumor-selective pro-apoptotic amplification mechanism that could complement or potentially surpass cytostatic strategies such as CDK4/6 inhibition covered in Portman et al.

## Conclusions

In this paper, a new unified therapeutic theory is presented that identifies novel master regulators of apoptosis as targets for treating cancer regardless of which organ the cancer lies in. This was discovered through a biochemical flowsheet of a universal apoptosis network comprising approximately 100 pathways (80% activation and 20% inhibition) developed in this work, based on a critical analysis of 172 scientific publications that considered all the complex interactions between proteins and regulatory RNAs. Using this comprehensive apoptosis network, three types of cancer were identified, based on the malfunction of a certain set of proteins and regulatory RNAs, irrespective of the organ in which the cancer is located. They are: Cancer Type 1, in which cancer cells lack either a functional (a) P14ARF gene, or (b) a P53 gene; Cancer Type 2, in which cancer cells lack a functional DINO lncRNA; and Cancer Type 3, in which cancer cells have abnormally high MDM2 protein activity. A biochemical flowsheet for each type of cancer was developed in this work and through that, the following therapeutic targets were discovered: (1) a universal therapeutic target for all three types of cancer, identified as the HuR cleavage inducer, (2) one specific therapeutic target for Cancer Type 2, which is the DINO lncRNA promoter, crucial to the proper function of the antioxidant activator protein P53, and (3) one specific target for Cancer Type 3, which is Mir-125b, a microRNA that is also involved in the antioxidant response of the cell. These are described in Joseph [168, 169].

Additionally, a comprehensive new biochemical flowsheet of the mechanistic pathway for the shuttling of antioxidant-producing HuR protein outside of the nucleus was developed, along with a new comparative biochemical flowsheet analyzing the differences in the response of HuR to sustainable and lethal cell damage. This work paves the way to treating the 3 types of cancer using one universal specific target, as well as treating cancers #2 and #3 with their own specific target.

### Biological complexity, experimental validation, and translational considerations

#### Biological complexity

The unified apoptosis theory presented in this work is intentionally systems-level and integrative, and as such necessarily engages with a degree of biological complexity that exceeds that of single-pathway or single-target models. Apoptosis, oxidative stress responses, and oncogenic survival mechanisms are not linear processes but highly interconnected regulatory networks involving proteins, microRNAs, and long noncoding RNAs. The complexity reflects the biological reality of cell death regulation rather than a weakness of the framework. The primary objective of this study is not to simplify apoptosis by reduction, but to organize existing mechanistic knowledge into a coherent, tractable, and logically unified network capable of generating testable predictions.

#### Experimental validation

This work is positioned as a theory-forming and hypothesis-generating contribution. All individual molecular interactions and regulatory mechanisms included in the unified apoptosis network have been previously demonstrated experimentally and are supported by peer-reviewed literature. The novelty of the present study lies in the integration of these experimentally validated components into a single universal framework that explains cancer survival and apoptotic failure across tissue types. The resulting classification of cancers into three mechanistically defined families, as well as the identification of context-dependent master regulators (HuR, miR-125b, and the DINO promoter), generates clear experimental hypotheses that can be directly tested in future in vitro, in vivo, and translational studies.

#### Translational challenges

Finally, translational challenges are inherent to any systems-level therapeutic framework in oncology. Importantly, however, the unified apoptosis theory directly addresses a major translational bottleneck in current cancer treatment: the reliance on organ-based classification rather than molecular apoptotic competency. By shifting the focus from tissue origin to conserved apoptotic malfunction states, this framework is intended to facilitate more rational patient stratification and therapeutic targeting. The proposed interventions—such as selective induction of HuR cleavage, inhibition of miR-125b in MDM2-amplified cancers, or reactivation of the DINO promoter—are explicitly designed to be adaptable to existing pharmacological, epigenetic, and RNA-targeting technologies, thereby providing a practical bridge between mechanistic insight and clinical application.

### Tumor heterogeneity, compensatory survival mechanisms, systemic toxicity, selectivity, and translational perspective

The unified apoptosis theory presented in this work necessarily operates within the context of biological complexity, tumor heterogeneity, and translational constraints that characterize modern oncology. These challenges are acknowledged here not as contradictions to the proposed framework, but as conditions that motivated its development.

#### Tumor heterogeneity

Tumor heterogeneity is a well-established barrier to effective cancer therapy and has historically undermined organ-based and histopathological cancer classifications. In this work, heterogeneity is explicitly incorporated at the mechanistic level by stratifying cancers according to the functional integrity of core apoptotic regulators rather than tissue of origin. The classification into three cancer families—defined by defects in P53/P14ARF signaling, DINO lncRNA function, or abnormally high MDM2 protein activity—reflects molecular heterogeneity in apoptotic competency rather than phenotypic diversity. By anchoring therapeutic strategy to apoptotic control nodes that are functionally decisive for cell survival, this framework aims to reduce the clinical ambiguity introduced by inter- and intra-tumour variability.

#### Compensatory survival mechanisms

Cancer cells frequently evade therapy through compensatory survival pathways, feedback loops, and redundancy within apoptotic regulators, particularly among BCL-2 family members, RNA-binding proteins, and microRNA networks. Rather than assuming linear pathway dominance, the unified apoptosis network explicitly integrates these adaptive mechanisms. Feedback loops involving HuR, NF-κB, Akt signaling, antiapoptotic BCL-2 proteins, and microRNA-mediated buffering are incorporated into the model to reflect realistic cellular behavior. The therapeutic strategies proposed in this work, therefore, target master regulatory nodes—such as HuR cleavage induction, miR-125b inhibition, or DINO promoter reactivation—that exert control across multiple survival pathways simultaneously, thereby limiting the capacity for single-pathway compensation and adaptive resistance.

#### Systemic toxicity and selectivity

A major concern in apoptosis-based therapies is the risk of systemic toxicity arising from indiscriminate activation of cell death pathways. Importantly, the present framework does not advocate global or non-selective induction of apoptosis. Instead, it emphasizes context-dependent regulators whose activity, localization, or expression is markedly altered in malignant cells relative to healthy tissues. For example, cytoplasmic overexpression and aberrant shuttling of HuR are disproportionately enriched in cancer cells, providing a mechanistic basis for tumor-biased apoptotic induction through HuR cleavage. Similarly, interventions targeting miR-125b or the DINO promoter are only relevant in specific molecular contexts and are not expected to disrupt apoptosis homeostasis in normal tissues. Thus, the model prioritizes therapeutic selectivity based on differential apoptotic wiring rather than systemic activation of death programs.

#### Translational perspective

This work is intended as a theory-forming and hypothesis-generating contribution that integrates existing experimental evidence into a unified systems-level model of apoptosis. While clinical translation will require rigorous experimental validation, biomarker stratification, and therapeutic optimization, the framework presented here provides a rational foundation for such efforts. By shifting the focus from organ-specific treatment paradigms to mechanistically grounded apoptotic states, this theory aims to simplify—not complicate—the translational pathway toward targeted cancer therapy.

In summary, tumor heterogeneity, compensatory survival mechanisms, and toxicity are not external obstacles to the unified apoptosis theory, but central features that it was explicitly designed to address. The integration of apoptotic complexity into a coherent, testable network represents a necessary step toward rational, mechanism-based cancer treatment strategies.

## Future directions

The unified apoptosis framework developed in this work opens several important avenues for future investigation, spanning translational oncology, therapeutic development, and fundamental disease biology. The following are the topics that our group is working on as a continuation of the current study.

First, a critical next step is the experimental validation and pharmacological development of the therapeutic strategies proposed herein. These include (i) the controlled induction of HuR cleavage in cancer cells lacking functional p53 activity, (ii) epigenetic reactivation of the DINO promoter in DINO-deficient tumours and (iii) targeted inhibition of microRNA-125b in cancers characterized by abnormally high MDM2 protein activity. Our group’s ongoing studies aim to identify small molecules, antisense oligonucleotides, or epigenetic modulators capable of selectively engaging these targets while minimizing toxicity to healthy tissues.

Second, further refinement of cancer classification using this apoptosis-based framework may improve precision oncology approaches. Since the three cancer types identified in this work are defined independently of tissue origin, large-scale genomic and transcriptomic datasets may be leveraged to retrospectively classify tumours according to p53 status, DINO integrity, and MDM2 amplification. This could enable stratification of patients for mechanism-guided therapies rather than organ-specific treatment paradigms.

Third, the universal apoptosis network described here provides a conceptual scaffold for extending these findings beyond oncology. Dysregulation of apoptosis and translational control is a hallmark not only of cancer, but also of neurodegenerative diseases and age-related disorders. Our group’s follow-up work will explore whether the master regulators identified in this study—particularly HuR-mediated translational control and p53-dependent stress responses—also govern cell fate decisions in neurodegenerative contexts, such as neurodegeneration linked to airway defense mechanisms, as seen in Sato et al. [170].

In this regard, the first author of this study has previously identified translational control mechanisms, such as the deamidation-dependent regulation of the translational repressor 4E-BP2, as fundamental drivers of neurodegeneration in Joseph [171] and Joseph [172]. Investigating whether the apoptotic master regulators elucidated here intersect with these translational checkpoints may reveal shared molecular denominators between cancer and neurodegenerative diseases. Such convergence would suggest that apoptosis regulation operates as a broader biological axis influencing cell survival across diverse pathological states.

Finally, our group’s further studies aim to computationally model the universal apoptosis network outlined in this work. Systems-level modelling may help predict network behaviour under different genetic perturbations, oxidative stress levels, and therapeutic interventions, thereby accelerating hypothesis testing and guiding experimental design.

Together, these future directions underscore the potential of this unified therapeutic theory not only to reshape cancer treatment strategies but also to inform a broader understanding of cell fate regulation in human disease.

## References

[1] Hajdu SI. A note from history: landmarks in history of cancer, part 1. Cancer. 2011;117:1097–102. 20960499 10.1002/cncr.25553

[2] Kandoth C, McLellan MD, Vandin F, Ye K, Niu B, Lu C, et al. Mutational landscape and significance across 12 major cancer types. Nature. 2013;502:333–9. 24132290 10.1038/nature12634 PMC3927368

[3] André F, Rassy E, Marabelle A, Michiels S, Besse B. Forget lung, breast or prostate cancer: why tumour naming needs to change. Nature. 2024;626:26–29. 38347121 10.1038/d41586-024-00216-3

[4] Mitchell E, Pham MH, Clay A, Sanghvi R, Williams N, Pietsch S, ... Stratton MR. The long-term effects of chemotherapy on normal blood cells. Nat Genet. 2025;57:1684–94. 10.1038/s41588-025-02234-x PMC12283364 40596443

[5] Kim YS, Tang PW, Welles JE, Pan W, Javed Z, Elhaw AT, et al. HuR-dependent SOD2 protein synthesis is an early adaptation to anchorage-independence. Redox Biol. 2022;53:102329. 35594792 10.1016/j.redox.2022.102329 PMC9121325

[6] Liu D, Xu Y. p53, oxidative stress, and aging. Antioxid Redox Signal. 2011;15:1669–78. 21050134 10.1089/ars.2010.3644 PMC3151427

[7] Pelullo M, Savi D, Quattrucci S, Cimino G, Pizzuti A, Screpanti I, et al. miR-125b/NRF2/HO-1 axis is involved in protection against oxidative stress of cystic fibrosis: a pilot study. Exp Therapeutic Med. 2021;21:585. 10.3892/etm.2021.10017 PMC8027740 33850557

[8] Hinman MN, Lou H. Diverse molecular functions of Hu proteins. Cell Mol Life Sci. 2008;65:3168–81. 18581050 10.1007/s00018-008-8252-6 PMC2580827

[9] Gorospe M. HuR in the mammalian genotoxic response: post-transcriptional multitasking. Cell Cycle. 2003;2:411–3. 12963828

[10] Wang W, Furneaux H, Cheng H, Caldwell MC, Hutter D, Liu Y, et al. HuR regulates p21 mRNA stabilization by UV light. Mol Cell Biol. 2000;20:760–9. 10629032 10.1128/mcb.20.3.760-769.2000 PMC85192

[11] Deng C, Zhang P, Harper JW, Elledge SJ, Leder P. Mice lacking p21CIP1/WAF1 undergo normal development, but are defective in G1 checkpoint control. Cell. 1995;82:675–84. 7664346 10.1016/0092-8674(95)90039-x

[12] Qi S, Calvi BR. Different cell cycle modifications repress apoptosis at different steps independent of developmental signaling in Drosophila. Mol Biol Cell. 2016;27:1885–97. 27075174 10.1091/mbc.E16-03-0139 PMC4907722

[13] Bertoli C, Skotheim JM, De Bruin RA. Control of cell cycle transcription during G1 and S phases. Nat Rev Mol Cell Biol. 2013;14:518–28. 23877564 10.1038/nrm3629 PMC4569015

[14] Lal A, Kawai T, Yang X, Mazan-Mamczarz K, Gorospe M. Antiapoptotic function of RNA-binding protein HuR effected through prothymosin α. EMBO J. 2005;24:1852–62. 15861128 10.1038/sj.emboj.7600661 PMC1142594

[15] Fan XC, Steitz JA. HNS, a nuclear-cytoplasmic shuttling sequence in HuR. Proc Natl Acad Sci. 1998;95:15293–8. 9860962 10.1073/pnas.95.26.15293 PMC28036

[16] Jiang X, Kim HE, Shu H, Zhao Y, Zhang H, Kofron J, et al. Distinctive roles of PHAP proteins and prothymosin-α in a death regulatory pathway. Science. 2003;299:223–6. 12522243 10.1126/science.1076807

[17] Abdelmohsen K, Pullmann R, Lal A, Kim HH, Galban S, Yang X, et al. Phosphorylation of HuR by Chk2 regulates SIRT1 expression. Mol cell. 2007;25:543–57. 17317627 10.1016/j.molcel.2007.01.011 PMC1986740

[18] Vaziri H, Dessain SK, Eaton EN, Imai SI, Frye RA, Pandita TK, et al. hSIR2SIRT1 functions as an NAD-dependent p53 deacetylase. Cell. 2001;107:149–59. 11672523 10.1016/s0092-8674(01)00527-x

[19] Luo J, Nikolaev AY, Imai SI, Chen D, Su F, Shiloh A, et al. Negative control of p53 by Sir2α promotes cell survival under stress. Cell. 2001;107:137–48. 11672522 10.1016/s0092-8674(01)00524-4

[20] Abdelmohsen K, Lal A, Kim HH, Gorospe M. Posttranscriptional orchestration of an anti-apoptotic program by HuR. Cell cycle. 2007;6:1288–92. 17534146 10.4161/cc.6.11.4299

[21] Miyashita TRJC, Reed JC. Tumor suppressor p53 is a direct transcriptional activator of the human bax gene. Cell. 1995;80:293–300. 7834749 10.1016/0092-8674(95)90412-3

[22] Fletcher JI, Meusburger S, Hawkins CJ, Riglar DT, Lee EF, Fairlie WD, et al. Apoptosis is triggered when prosurvival Bcl-2 proteins cannot restrain Bax. Proc Natl Acad Sci. 2008;105:18081–7. 18981409 10.1073/pnas.0808691105 PMC2577705

[23] Aubrey BJ, Kelly GL, Janic A, Herold MJ, Strasser A. How does p53 induce apoptosis and how does this relate to p53-mediated tumour suppression? Cell Death Differ. 2018;25:104–13. 29149101 10.1038/cdd.2017.169 PMC5729529

[24] Rathmell JC, Lindsten T, Zong WX, Cinalli RM, Thompson CB. Deficiency in Bak and Bax perturbs thymic selection and lymphoid homeostasis. Nat Immunol. 2002;3:932–9. 12244308 10.1038/ni834

[25] Cohen HY, Lavu S, Bitterman KJ, Hekking B, Imahiyerobo TA, Miller C, et al. Acetylation of the C terminus of Ku70 by CBP and PCAF controls Bax-mediated apoptosis. Molecular cell. 2004;13:627–38. 15023334 10.1016/s1097-2765(04)00094-2

[26] Oda E, Ohki R, Murasawa H, Nemoto J, Shibue T, Yamashita T, et al. Noxa, a BH3-only member of the Bcl-2 family and candidate mediator of p53-induced apoptosis. Science. 2000;288:1053–8. 10807576 10.1126/science.288.5468.1053

[27] Huang DC, Strasser A. BH3-only proteins—essential initiators of apoptotic cell death. Cell. 2000;103:839–42. 11136969 10.1016/s0092-8674(00)00187-2

[28] Antignani A, Youle RJ. How do Bax and Bak lead to permeabilization of the outer mitochondrial membrane? Curr Opin Cell Biol. 2006;18:685–9. 17046225 10.1016/j.ceb.2006.10.004

[29] Czabotar PE, Westphal D, Dewson G, Ma S, Hockings C, Fairlie WD, et al. Bax crystal structures reveal how BH3 domains activate Bax and nucleate its oligomerization to induce apoptosis. Cell. 2013;152:519–31. 23374347 10.1016/j.cell.2012.12.031

[30] Dewson G, Kratina T, Sim HW, Puthalakath H, Adams JM, Colman PM, et al. To trigger apoptosis, Bak exposes its BH3 domain and homodimerizes via BH3: groove interactions. Mol Cell. 2008;30:369–80. 18471982 10.1016/j.molcel.2008.04.005

[31] Moldoveanu T, Liu Q, Tocilj A, Watson M, Shore G, Gehring K. The X-ray structure of a BAK homodimer reveals an inhibitory zinc binding site. Mol Cell. 2006;24:677–88. 17157251 10.1016/j.molcel.2006.10.014

[32] Dewson G, Kratina T, Czabotar P, Day CL, Adams JM, Kluck RM. Bak activation for apoptosis involves oligomerization of dimers via their α6 helices. Mol Cell. 2009;36:696–703. 19941828 10.1016/j.molcel.2009.11.008

[33] Dewson G, Ma S, Frederick P, Hockings C, Tan I, Kratina T, et al. Bax dimerizes via a symmetric BH3: groove interface during apoptosis. Cell Death Differ. 2012;19:661–70. 22015607 10.1038/cdd.2011.138 PMC3307980

[34] Desagher S, Osen-Sand A, Nichols A, Eskes R, Montessuit S, Lauper S, et al. Bid-induced conformational change of Bax is responsible for mitochondrial cytochrome c release during apoptosis. J Cell Biol. 1999;144:891–901. 10085289 10.1083/jcb.144.5.891 PMC2148190

[35] Li H, Zhu H, Xu CJ, Yuan J. Cleavage of BID by caspase 8 mediates the mitochondrial damage in the Fas pathway of apoptosis. Cell. 1998;94:491–501. 9727492 10.1016/s0092-8674(00)81590-1

[36] Beaudouin J, Liesche C, Aschenbrenner S, Hörner M, Eils R. Caspase-8 cleaves its substrates from the plasma membrane upon CD95-induced apoptosis. Cell Death Differ. 2013;20:599–610. 23306557 10.1038/cdd.2012.156 PMC3595484

[37] Kischkel FC, Hellbardt S, Behrmann I, Germer M, Pawlita M, Krammer PH, et al. Cytotoxicity-dependent APO-1 (Fas/CD95)-associated proteins form a death-inducing signaling complex (DISC) with the receptor. EMBO J. 1995;14:5579–88. 8521815 10.1002/j.1460-2075.1995.tb00245.x PMC394672

[38] Jeong EJ, Bang S, Lee TH, Park YI, Sim WS, Kim KS. The solution structure of FADD death domain: structural basis of death domain interactions of Fas and FADD. J Biol Chem. 1999;274:16337–42. 10347191 10.1074/jbc.274.23.16337

[39] JP M. FLICE is activated by association with the CD95 death-inducing signaling complex (DISC). EMBO J. 1997;81:935–45. 10.1093/emboj/16.10.2794 PMC1169888 9184224

[40] Hughes MA, Harper N, Butterworth M, Cain K, Cohen GM, MacFarlane M. Reconstitution of the death-inducing signaling complex reveals a substrate switch that determines CD95-mediated death or survival. Mol Cell. 2009;35:265–79. 19683492 10.1016/j.molcel.2009.06.012

[41] Scaffidi C, Medema JP, Krammer PH, Peter ME. FLICE is predominantly expressed as two functionally active isoforms, caspase-8/a and caspase-8/b. J Biol Chem. 1997;272:26953–8. 9341131 10.1074/jbc.272.43.26953

[42] Lavrik I, Krueger A, Schmitz I, Baumann S, Weyd H, Krammer PH, et al. The active caspase-8 heterotetramer is formed at the CD95 DISC. Cell Death Differ. 2003;10:144–5. 12655304 10.1038/sj.cdd.4401156

[43] Kataoka T, Tschopp J. N-terminal fragment of c-FLIP (L) processed by caspase 8 specifically interacts with TRAF2 and induces activation of the NF-κB signaling pathway. Mol Cell Biol. 2004;24:2627–36. 15024054 10.1128/MCB.24.7.2627-2636.2004 PMC371124

[44] Han JH, Park J, Kang TB, Lee KH. Regulation of caspase-8 activity at the crossroads of pro-inflammation and anti-inflammation. Int J Mol Sci. 2021;22:3318. 33805003 10.3390/ijms22073318 PMC8036737

[45] Eskes R, Desagher S, Antonsson B, Martinou JC. Bid induces the oligomerization and insertion of Bax into the outer mitochondrial membrane. Mol Cell Biol. 2000;20:929–35. 10629050 10.1128/mcb.20.3.929-935.2000 PMC85210

[46] Cartron PF, Gallenne T, Bougras G, Gautier F, Manero F, Vusio P, et al. The first α helix of Bax plays a necessary role in its ligand-induced activation by the BH3-only proteins Bid and PUMA. Mol Cell. 2004;16:807–18. 15574335 10.1016/j.molcel.2004.10.028

[47] Cartron PF, Priault M, Oliver L, Meflah K, Manon S, Vallette FM. The N-terminal end of Bax contains a mitochondrial-targeting signal. J Biol Chem. 2003;278:11633–41. 12529375 10.1074/jbc.M208955200

[48] Nechushtan, A, Smith, CL, Hsu, YT, & Youle, RJ. Conformation of the Bax C-terminus regulates subcellular location and cell death. EMBO Journal.1999; 10.1093/emboj/18.9.2330 PMC1171316 10228148

[49] Sax JK, Fei P, Murphy ME, Bernhard E, Korsmeyer SJ, El-Deiry WS. BID regulation by p53 contributes to chemosensitivity. Nat Cell Biol. 2002;4:842–9. 12402042 10.1038/ncb866

[50] Vogler M, Braun Y, Smith VM, Westhoff MA, Pereira RS, Pieper NM, et al. The BCL2 family: from apoptosis mechanisms to new advances in targeted therapy. Signal Transduct Target Ther. 2025;10:91. 40113751 10.1038/s41392-025-02176-0 PMC11926181

[51] Ishimaru D, Ramalingam S, Sengupta TK, Bandyopadhyay S, Dellis S, Tholanikunnel BG, et al. Regulation of Bcl-2 expression by HuR in HL60 leukemia cells and A431 carcinoma cells. Mol Cancer Res. 2009;7:1354–66. 19671677 10.1158/1541-7786.MCR-08-0476

[52] Chiou JT, Lee YC, Huang CH, Shi YJ, Wang LJ, Chang LS. Autophagic HuR mRNA degradation induces survivin and MCL1 downregulation in YM155-treated human leukemia cells. Toxicol Appl Pharmacol. 2020;387:114857. 31837377 10.1016/j.taap.2019.114857

[53] Lomonosova E, Chinnadurai G. BH3-only proteins in apoptosis and beyond: an overview. Oncogene. 2008;27:S2–S19. 19641503 10.1038/onc.2009.39 PMC2928556

[54] Fiebig AA, Zhu W, Hollerbach C, Leber B, Andrews DW. Bcl-XL is qualitatively different from and ten times more effective than Bcl-2 when expressed in a breast cancer cell line. BMC Cancer. 2006;6:1–15. 16928273 10.1186/1471-2407-6-213 PMC1560389

[55] Oliver FJ, de la Rubia G, Rolli V, Ruiz-Ruiz MC, de Murcia G, Ménissier-de Murcia J. Importance of poly (ADP-ribose) polymerase and its cleavage in apoptosis: lesson from an uncleavable mutant. J Biol Chem. 1998;273:33533–9. 9837934 10.1074/jbc.273.50.33533

[56] Billen LP, Kokoski CL, Lovell JF, Leber B, Andrews DW. Bcl-XL inhibits membrane permeabilization by competing with Bax. PLoS Biol. 2008;6:e147. 18547146 10.1371/journal.pbio.0060147 PMC2422857

[57] Leitl KD, Sperl LE, Hagn F. Preferred inhibition of pro-apoptotic Bak by BclxL via a two-step mechanism. Cell Rep. 2024;43:114526. 10.1016/j.celrep.2024.114526 39046879

[58] Ku B, Liang C, Jung JU, Oh BH. Evidence that inhibition of BAX activation by BCL-2 involves its tight and preferential interaction with the BH3 domain of BAX. Cell Res. 2011;21:627–41. 21060336 10.1038/cr.2010.149 PMC3343310

[59] Reynolds JE, Yang T, Qian L, Jenkinson JD, Zhou P, Eastman A, et al. Mcl-1, a member of the Bcl-2 family, delays apoptosis induced by c-Myc overexpression in Chinese hamster ovary cells. Cancer Res. 1994;54:6348–52. 7987827

[60] Talwar S, Jin J, Carroll B, Liu A, Gillespie MB, Palanisamy V. Caspase-mediated cleavage of RNA-binding protein HuR regulates c-Myc protein expression after hypoxic stress. J Biol Chem. 2011;286:32333–43. 21795698 10.1074/jbc.M111.255927 PMC3173192

[61] Askew DS, Ashmun RA, Simmons BC, Cleveland JL. Constitutive c-myc expression in an IL-3-dependent myeloid cell line suppresses cell cycle arrest and accelerates apoptosis. Oncogene. 1991;6:1915–22. 1923514

[62] Evan GI, Wyllie AH, Gilbert CS, Littlewood TD, Land H, Brooks M, et al. Induction of apoptosis in fibroblasts by c-myc protein. Cell. 1992;69:119–28. 1555236 10.1016/0092-8674(92)90123-t

[63] Brizzi MF, Garbarino G, Rossi PR, Pagliardi GL, Arduino C, Avanzi GC, et al. Interleukin 3 stimulates proliferation and triggers endothelial-leukocyte adhesion molecule 1 gene activation of human endothelial cells. J Clin Investig. 1993;91:2887–92. 7685775 10.1172/JCI116534 PMC443359

[64] Miller DM, Thomas SD, Islam A, Muench D, Sedoris K. c-Myc and cancer metabolism. Clin Cancer Res. 2012;18:5546–53. 23071356 10.1158/1078-0432.CCR-12-0977 PMC3505847

[65] Wu D, Kaan HYK, Zheng X, Tang X, He Y, Vanessa Tan Q, et al. Structural basis of Ornithine Decarboxylase inactivation and accelerated degradation by polyamine sensor Antizyme1. Sci Rep. 2015;5:14738. 26443277 10.1038/srep14738 PMC4595762

[66] Zindy F, Eischen CM, Randle DH, Kamijo T, Cleveland JL, Sherr CJ, et al. Myc signaling via the ARF tumor suppressor regulates p53-dependent apoptosis and immortalization. Genes Dev. 1998;12:2424–33. 9694806 10.1101/gad.12.15.2424 PMC317045

[67] Eischen CM, Weber JD, Roussel MF, Sherr CJ, Cleveland JL. Disruption of the ARF–Mdm2–p53 tumor suppressor pathway in Myc-induced lymphomagenesis. Genes Dev. 1999;13:2658–69. 10541552 10.1101/gad.13.20.2658 PMC317106

[68] Kamijo T, Zindy F, Roussel MF, Quelle DE, Downing JR, Ashmun RA, et al. Tumor suppression at the mouse INK4a locus mediated by the alternative reading frame product p19 ARF. Cell. 1997;91:649–59. 9393858 10.1016/s0092-8674(00)80452-3

[69] Kamijo T, Weber JD, Zambetti G, Zindy F, Roussel MF, Sherr CJ. Functional and physical interactions of the ARF tumor suppressor with p53 and Mdm2. Proc Natl Acad Sci. 1998;95:8292–7. 9653180 10.1073/pnas.95.14.8292 PMC20969

[70] Momand J, Zambetti GP, Olson DC, George D, Levine AJ. The mdm-2 oncogene product forms a complex with the p53 protein and inhibits p53-mediated transactivation. cell. 1992;69:1237–45. 1535557 10.1016/0092-8674(92)90644-r

[71] Pomerantz J, Schreiber-Agus N, Liégeois NJ, Silverman A, Alland L, Chin L, et al. The Ink4a tumor suppressor gene product, p19Arf, interacts with MDM2 and neutralizes MDM2’s inhibition of p53. Cell. 1998;92:713–23. 9529248 10.1016/s0092-8674(00)81400-2

[72] Ghosh M, Aguila HL, Michaud J, Ai Y, Wu MT, Hemmes A, et al. Essential role of the RNA-binding protein HuR in progenitor cell survival in mice. J Clin Investig 2009;119. 10.1172/JCI38263 PMC2786787 19884656

[73] Amir S, Ma AH, Shi XB, Xue L, Kung HJ, deVere White RW. Oncomir miR-125b suppresses p14ARF to modulate p53-dependent and p53-independent apoptosis in prostate cancer. PloS one. 2013;8:e61064. 23585871 10.1371/journal.pone.0061064 PMC3621663

[74] Kale J, Osterlund EJ, Andrews DW. BCL-2 family proteins: changing partners in the dance towards death. Cell Death Differ. 2018;25:65–80. 29149100 10.1038/cdd.2017.186 PMC5729540

[75] Yu J, Zhang L, Hwang PM, Kinzler KW, Vogelstein B. PUMA induces the rapid apoptosis of colorectal cancer cells. Mol Cell. 2001;7:673–82. 11463391 10.1016/s1097-2765(01)00213-1

[76] Nakano K, Vousden KH. PUMA, a novel proapoptotic gene, is induced by p53. Mol Cell. 2001;7:683–94. 11463392 10.1016/s1097-2765(01)00214-3

[77] Chen L, Willis SN, Wei A, Smith BJ, Fletcher JI, Hinds MG, et al. Differential targeting of prosurvival Bcl-2 proteins by their BH3-only ligands allows complementary apoptotic function. Mol Cell. 2005;17:393–403. 15694340 10.1016/j.molcel.2004.12.030

[78] Werner AB, de Vries E, Tait SW, Bontjer I, Borst J. Bcl-2 family member Bfl-1/A1 sequesters truncated bid to inhibit its collaboration with pro-apoptotic Bak or Bax. J Biol Chem. 2002;277:22781–8. 11929871 10.1074/jbc.M201469200

[79] Sharma K, Vu TT, Naseri M, Nakajima W, Zhan K, Harada H. p53-independent Noxa induction by cisplatin is regulated by ATF3/ATF4 in HNSCC cells. Cancer Res. 2017;77:2127–2127. 10.1002/1878-0261.12172 PMC5983129 29352505

[80] Jiang P, Du W, Heese K, Wu M. The Bad guy cooperates with good cop p53: Bad is transcriptionally up-regulated by p53 and forms a Bad/p53 complex at the mitochondria to induce apoptosis. Mol Cell Biol. 2006;26:9071–82. 17000778 10.1128/MCB.01025-06 PMC1636833

[81] Brunet A, Sweeney LB, Sturgill JF, Chua KF, Greer PL, Lin Y, et al. Stress-dependent regulation of FOXO transcription factors by the SIRT1 deacetylase. Science. 2004;303:2011–5. 14976264 10.1126/science.1094637

[82] Brunet A, Bonni A, Zigmond MJ, Lin MZ, Juo P, Hu LS, et al. Akt promotes cell survival by phosphorylating and inhibiting a Forkhead transcription factor. Cell. 1999;96:857–68. 10102273 10.1016/s0092-8674(00)80595-4

[83] Morris BJ, Willcox DC, Donlon TA, Willcox BJ. FOXO3: a major gene for human longevity-a mini-review. Gerontology. 2015;61:515–25. 25832544 10.1159/000375235 PMC5403515

[84] Friesen C, Herr I, Krammer PH, Debatin KM. Involvement of the CD95 (APO–1/Fas) receptor/ligand system in drug–induced apoptosis in leukemia cells. Nat Med. 1996;2:574–7. 8616718 10.1038/nm0596-574

[85] Dijkers PF, Birkenkamp KU, Lam EWF, Thomas NSB, Lammers JWJ, Koenderman L, et al. FKHR-L1 can act as a critical effector of cell death induced by cytokine withdrawal: protein kinase B–enhanced cell survival through maintenance of mitochondrial integrity. J Cell Biol. 2002;156:531–42. 11815629 10.1083/jcb.200108084 PMC2173339

[86] Sunters A, de Mattos SF, Stahl M, Brosens JJ, Zoumpoulidou G, Saunders CA, et al. FoxO3a transcriptional regulation of Bim controls apoptosis in paclitaxel-treated breast cancer cell lines. J Biol Chem. 2003;278:49795–805. 14527951 10.1074/jbc.M309523200

[87] O’Connor L, Strasser A, O'Reilly LA, Hausmann G, Adams JM, Cory S, et al. Bim: a novel member of the Bcl‐2 family that promotes apoptosis. The EMBO J 1998;17:384–95. 10.1093/emboj/17.2.384 PMC1170389 9430630

[88] Chi X, Nguyen D, Pemberton JM, Osterlund EJ, Liu Q, Brahmbhatt H, et al. The carboxyl-terminal sequence of bim enables bax activation and killing of unprimed cells. Elife. 2020;9:e44525. 31976859 10.7554/eLife.44525 PMC6980855

[89] Sarosiek KA, Chi X, Bachman JA, Sims JJ, Montero J, Patel L, et al. BID preferentially activates BAK while BIM preferentially activates BAX, affecting chemotherapy response. Mol Cell. 2013;51:751–65. 24074954 10.1016/j.molcel.2013.08.048 PMC4164233

[90] Yang MC, Lin RW, Huang SB, Huang SY, Chen WJ, Wang S, et al. Bim directly antagonizes Bcl-xl in doxorubicin-induced prostate cancer cell apoptosis independently of p53. Cell Cycle. 2016;15:394–402. 26694174 10.1080/15384101.2015.1127470 PMC4943702

[91] Han J, Goldstein LA, Hou W, Gastman BR, Rabinowich H. Regulation of mitochondrial apoptotic events by p53-mediated disruption of complexes between antiapoptotic Bcl-2 members and Bim. J Biol Chem. 2010;285:22473–83. 20404322 10.1074/jbc.M109.081042 PMC2903343

[92] Gomez-Bougie P, Bataille R, Amiot M. The imbalance between Bim and Mcl-1 expression controls the survival of human myeloma cells. Eur J Immunol. 2004;34:3156–64. 15459900 10.1002/eji.200424981

[93] Czabotar PE, Lee EF, van Delft MF, Day CL, Smith BJ, Huang DC, et al. Structural insights into the degradation of Mcl-1 induced by BH3 domains. Proc Natl Acad Sci. 2007;104:6217–22. 17389404 10.1073/pnas.0701297104 PMC1851040

[94] Hagenbuchner J, Kiechl-Kohlendorfer U, Obexer P, Ausserlechner MJ. A novel Mcl1 variant inhibits apoptosis via increased Bim sequestration. Oncotarget. 2013;4:1241. 23872733 10.18632/oncotarget.1147 PMC3787154

[95] Domina AM, Vrana JA, Gregory MA, Hann SR, Craig RW. MCL1 is phosphorylated in the PEST region and stabilized upon ERK activation in viable cells, and at additional sites with cytotoxic okadaic acid or taxol. Oncogene. 2004;23:5301–15. 15241487 10.1038/sj.onc.1207692

[96] Germain M, Milburn J, Duronio V. MCL-1 inhibits BAX in the absence of MCL-1/BAX Interaction. J Biol Chem. 2008;283:6384–92. 18089567 10.1074/jbc.M707762200

[97] Willis SN, Chen L, Dewson G, Wei A, Naik E, Fletcher JI, et al. Proapoptotic Bak is sequestered by Mcl-1 and Bcl-xL, but not Bcl-2, until displaced by BH3-only proteins. Genes Dev. 2005;19:1294–305. 15901672 10.1101/gad.1304105 PMC1142553

[98] Tran H, Brunet A, Grenier JM, Datta SR, Fornace Jr AJ, DiStefano PS et al. DNA repair pathway stimulated by the forkhead transcription factor FOXO3a through the Gadd45 protein. Science. 2002;296,530–4. 10.1126/science.1068712 11964479

[99] Chandramouly G,. Gadd45 in DNA demethylation and DNA repair. In *Gadd45 stress sensor genes* (pp. 55-67). Cham: Springer International Publishing 2022. 10.1007/978-3-030-94804-7_4 35505162

[100] Singh M, Martinez AR, Govindaraju S, Lee BS. HuR inhibits apoptosis by amplifying Akt signaling through a positive feedback loop. J Cell Physiol. 2013;228:182–9. 22674407 10.1002/jcp.24120 PMC3443509

[101] Mazan-Mamczarz K, Galbán S, de Silanes IL, Martindale JL, Atasoy U, Keene JD, et al. RNA-binding protein HuR enhances p53 translation in response to ultraviolet light irradiation. Proc Natl Acad Sci. 2003;100:8354–9. 12821781 10.1073/pnas.1432104100 PMC166233

[102] Ahuja D, Goyal A, Ray PS. Interplay between RNA-binding protein HuR and microRNA-125b regulates p53 mRNA translation in response to genotoxic stress. RNA Biol. 2016;13:1152–65. 27592685 10.1080/15476286.2016.1229734 PMC5100343

[103] Guha A, Ahuja D, Mandal SD, Parasar B, Deyasi K, Roy D, et al. Integrated regulation of HuR by translation repression and protein degradation determines pulsatile expression of p53 under DNA damage. Iscience. 2019;15:342–59. 31103853 10.1016/j.isci.2019.05.002 PMC6548907

[104] Goswami B, Ahuja D, Pastré D, Ray PS. p53 and HuR combinatorially control the biphasic dynamics of microRNA-125b in response to genotoxic stress. Commun Biol. 2023;6,110. 10.1038/s42003-023-04507-9 PMC9883498 36707647

[105] Donahue JM, Chang ET, Xiao L, Wang PY, Rao JN, Turner DJ, et al. The RNA-binding protein HuR stabilizes survivin mRNA in human oesophageal epithelial cells. Biochemical J. 2011;437:89–96. 10.1042/BJ20110028 21443519

[106] Levine AJ. p53: 800 million years of evolution and 40 years of discovery. Nat Rev Cancer. 2020;20:471–80. 32404993 10.1038/s41568-020-0262-1

[107] Adamkov M, Halasova E, Rajcani J, Bencat M, Vybohova D, Rybarova S, et al. Relation between expression pattern of p53 and survivin in cutaneous basal cell carcinomas. Med Sci Monit: Int Med J Exp Clin Res. 2011;17:BR74. 10.12659/MSM.881442 PMC3524735 21358596

[108] Hoffman WH, Biade S, Zilfou JT, Chen J, Murphy M. Transcriptional repression of the anti-apoptoticsurvivin gene by wild type p53. J Biol Chem. 2002;277:3247–57. 11714700 10.1074/jbc.M106643200

[109] Wasylishen AR, Lozano G. Attenuating the p53 pathway in human cancers: many means to the same end. Cold Spring Harb Perspect Med. 2016;6:a026211. 27329033 10.1101/cshperspect.a026211 PMC4968169

[110] Ma WJ, Chung S, Furneaux H. The Elav-like proteins bind to AU-rich elements and to the poly (A) tail of mRNA. Nucleic Acids Res. 1997;25:3564–9. 9278474 10.1093/nar/25.18.3564 PMC146929

[111] Brennan CM, Gallouzi IE, Steitz JA. Protein ligands to HuR modulate its interaction with target mRNAs in vivo. J Cell Biol. 2000;151:1–14. 11018049 10.1083/jcb.151.1.1 PMC2189805

[112] Herz HM, Garruss A, Shilatifard A. SET for life: biochemical activities and biological functions of SET domain-containing proteins. Trends Biochemical Sci. 2013;38:621–39. 10.1016/j.tibs.2013.09.004 PMC3941473 24148750

[113] Schmidt-Zachmann MS, Dargemont C, Kühn LC, Nigg EA. Nuclear export of proteins: the role of nuclear retention. Cell. 1993;74:493–504. 8348616 10.1016/0092-8674(93)80051-f

[114] Fornerod M, Ohno M, Yoshida M, Mattaj IW. CRM1 is an export receptor for leucine-rich nuclear export signals. Cell. 1997;90:1051–60. 9323133 10.1016/s0092-8674(00)80371-2

[115] Melchior F. Inhibition of nuclear-protein import by nonhydrolyzable analogs of gtp and identification of the small gtpase ran/tc4 as an essential transport factor (VOL 123, PG 1649, 1993). J Cell Biol. 1994;124:217–217. 10.1083/jcb.123.6.1649 PMC2290879 8276887

[116] Neville M, Stutz F, Lee L, Davis LI, Rosbash M. The importin-beta family member Crm1p bridges the interaction between Rev and the nuclear pore complex during nuclear export. Curr Biol. 1997;7:767–75. 9368759 10.1016/s0960-9822(06)00335-6

[117] Stutz F, Neville M, Rosbash M. Identification of a novel nuclear pore-associated protein as a functional target of the HIV-1 Rev protein in yeast. Cell. 1995;82:495–506. 7634338 10.1016/0092-8674(95)90438-7

[118] Waldmann I, Spillner C, Kehlenbach RH. The nucleoporin-like protein NLP1 (hCG1) promotes CRM1-dependent nuclear protein export. J Cell Sci. 2012;125:144–54. 22250199 10.1242/jcs.090316

[119] Chakravarti D, Hong R. SET-ting the stage for life and death. Cell. 2003;112:589–91. 12628178 10.1016/s0092-8674(03)00151-x

[120] Mazroui R, Di Marco S, Clair E, Von Roretz C, Tenenbaum SA, Keene JD, et al. Caspase-mediated cleavage of HuR in the cytoplasm contributes to pp32/PHAP-I regulation of apoptosis. J Cell Biol. 2008;180:113–27. 18180367 10.1083/jcb.200709030 PMC2213623

[121] Von Roretz C, Jin Lian X, Macri AM, Punjani N, Clair E, Drouin O, et al. Apoptotic-induced cleavage shifts HuR from being a promoter of survival to an activator of caspase-mediated apoptosis. Cell Death Differ. 2013;20:154–68. 22955946 10.1038/cdd.2012.111 PMC3524645

[122] Wu CC, Bratton SB. Caspase-9 swings both ways in the apoptosome. Mol Cell Oncol. 2017;4:e1281865. 28401186 10.1080/23723556.2017.1281865 PMC5383358

[123] Fadeel B, Ottosson A, Pervaiz S. Big wheel keeps on turning: apoptosome regulation and its role in chemoresistance. Cell Death Differ. 2008;15:443–52. 17975549 10.1038/sj.cdd.4402265

[124] Zhou M, Li Y, Hu Q, Bai XC, Huang W, Yan C, et al. Atomic structure of the apoptosome: mechanism of cytochrome c-and dATP-mediated activation of Apaf-1. Genes Dev. 2015;29:2349–61. 26543158 10.1101/gad.272278.115 PMC4691890

[125] Von Roretz C, Macri AM, Gallouzi IE. Transportin 2 regulates apoptosis through the RNA-binding protein HuR. J Biol Chem. 2011;286:25983–91. 21646354 10.1074/jbc.M110.216184 PMC3138312

[126] Rebane ANA, Aab A, Steitz JA. Transportins 1 and 2 are redundant nuclear import factors for hnRNP A1 and HuR. Rna. 2004;10:590–9. 15037768 10.1261/rna.5224304 PMC1370549

[127] Güttinger S, Mühlhäusser P, Koller-Eichhorn R, Brennecke J, Kutay U. Transportin2 functions as importin and mediates nuclear import of HuR. Proc Natl Acad Sci. 2004;101:2918–23. 14981248 10.1073/pnas.0400342101 PMC365720

[128] Van Der Giessen K, Gallouzi IE. Involvement of transportin 2–MEDIATED HuR import in muscle cell differentiation. Mol Biol Cell. 2007;18:2619–29. 17475777 10.1091/mbc.E07-02-0167 PMC1924833

[129] Kurosu T, Ohga N, Hida Y, Maishi N, Akiyama K, Kakuguchi W, et al. HuR keeps an angiogenic switch on by stabilising mRNA of VEGF and COX-2 in tumour endothelium. Br J Cancer. 2011;104:819–29. 21285980 10.1038/bjc.2011.20 PMC3048211

[130] Kim KJ, Li B, Winer J, Armanini M, Gillett N, Phillips HS, et al. Inhibition of vascular endothelial growth factor-induced angiogenesis suppresses tumour growth in vivo. Nature. 1993;362:841–4. 7683111 10.1038/362841a0

[131] Chandrasekharan NV, Simmons DL. The cyclooxygenases. Genome Biol. 2004;5:1–7. 10.1186/gb-2004-5-9-241 PMC522864 15345041

[132] Xu L, Stevens J, Hilton MB, Seaman S, Conrads TP, Veenstra TD, et al. COX-2 inhibition potentiates antiangiogenic cancer therapy and prevents metastasis in preclinical models. Sci Transl Med. 2014;6:242ra84–242ra84. 24964992 10.1126/scitranslmed.3008455 PMC6309995

[133] Durie D, Lewis SM, Liwak U, Kisilewicz M, Gorospe M, Holcik M. RNA-binding protein HuR mediates cytoprotection through stimulation of XIAP translation. Oncogene. 2011;30:1460–9. 21102524 10.1038/onc.2010.527 PMC3514411

[134] Scott FL, Denault JB, Riedl SJ, Shin H, Renatus M, Salvesen GS. XIAP inhibits caspase-3 and-7 using two binding sites: evolutionarily conserved mechanism of IAPs. EMBO J. 2005;24:645–55. 15650747 10.1038/sj.emboj.7600544 PMC548652

[135] Deveraux QL, Leo E, Stennicke HR, Welsh K, Salvesen GS, Reed JC. Cleavage of human inhibitor of apoptosis protein XIAP results in fragments with distinct specificities for caspases. The EMBO J. 1999;18:5242–51. 10.1093/emboj/18.19.5242 PMC1171595 10508158

[136] Nabors LB, Gillespie GY, Harkins L, King PH. HuR, a RNA stability factor, is expressed in malignant brain tumors and binds to adenine-and uridine-rich elements within the 3′ untranslated regions of cytokine and angiogenic factor mRNAs. Cancer Res. 2001;61:2154–61. 11280780

[137] Ferrari G, Cook BD, Terushkin V, Pintucci G, Mignatti P. Transforming growth factor-beta 1 (TGF-β1) induces angiogenesis through vascular endothelial growth factor (VEGF)-mediated apoptosis. J Cell Physiol. 2009;219:449–58. 19180561 10.1002/jcp.21706 PMC2749291

[138] Shi JUN, Wei PK. Interleukin-8: A potent promoter of angiogenesis in gastric cancer. Oncol Lett. 2016;11:1043–50. 26893688 10.3892/ol.2015.4035 PMC4734231

[139] Folkman, J (2003, April). Angiogenesis and apoptosis. In *Seminars in Cancer Biology* (Vol. 13, pp. 159-67). Academic Press. 10.1016/s1044-579x(02)00133-5 12654259

[140] Wu X, Ramesh R, Wang J, Zheng Y, Armaly AM, Wei L, et al. Small molecules targeting the RNA-binding protein HuR inhibit tumor growth in xenografts. J Medicinal Chem. 2023;66:2032–53. 10.1021/acs.jmedchem.2c01723 PMC10101218 36690437

[141] Wei L, Kim SH, Armaly AM, Aubé J, Xu L, Wu X. RNA-binding protein HuR inhibition induces multiple programmed cell death in breast and prostate cancer. Cell Commun Signal. 2024;22:1–14. 39627778 10.1186/s12964-024-01916-z PMC11613925

[142] Liu S, Huang Z, Tang A, Wu X, Aube J, Xu L et al. Inhibition of RNA-binding protein HuR reduces glomerulosclerosis in experimental nephritis. Clin Sci. 2020;134,1433–48. 10.1042/CS20200193 PMC8086301 32478392

[143] Dixon SJ, Patel DN, Welsch M, Skouta R, Lee ED, Hayano M, et al. Pharmacological inhibition of cystine–glutamate exchange induces endoplasmic reticulum stress and ferroptosis. elife. 2014;3:e02523. 24844246 10.7554/eLife.02523 PMC4054777

[144] Dixon SJ, Olzmann JA. The cell biology of ferroptosis. Nat Rev Mol Cell Biol. 2024;25:424–42. 38366038 10.1038/s41580-024-00703-5 PMC12187608

[145] Dixon SJ, Lemberg KM, Lamprecht MR, Skouta R, Zaitsev EM, Gleason CE, et al. Ferroptosis: an iron-dependent form of nonapoptotic cell death. Cell. 2012;149:1060–72. 22632970 10.1016/j.cell.2012.03.042 PMC3367386

[146] Yan Y, Teng H, Hang Q, Kondiparthi L, Lei G, Horbath A, et al. SLC7A11 expression level dictates differential responses to oxidative stress in cancer cells. Nat Commun. 2023;14:3673. 37339981 10.1038/s41467-023-39401-9 PMC10281978

[147] Lubos E, Loscalzo J, Handy DE. Glutathione peroxidase-1 in health and disease: from molecular mechanisms to therapeutic opportunities. Antioxid Redox Signal. 2011;15:1957–97. 10.1089/ars.2010.3586 PMC3159114 21087145

[148] Zhao Q, Su Y, Wang Z, Chen C, Wu T, Huang Y. Identification of glutathione (GSH)-independent glyoxalase III from Schizosaccharomyces pombe. BMC Evolut Biol. 2014;14:86. 10.1186/1471-2148-14-86 PMC4021431 24758716

[149] Tang JUN, ROSE RL, Chambers JE. Metabolism of organophosphorus and carbamate pesticides. In *Toxicology of organophosphate & carbamate compounds* (pp. 127-43). Academic Press 2006.

[150] Jiang L, Kon N, Li T, Wang SJ, Su T, Hibshoosh H, et al. Ferroptosis as a p53-mediated activity during tumour suppression. Nature. 2015;520:57–62. 25799988 10.1038/nature14344 PMC4455927

[151] Kataoka K, Nagata Y, Kitanaka A, Shiraishi Y, Shimamura T, Yasunaga JI, et al. Integrated molecular analysis of adult T cell leukemia/lymphoma. Nat Genet. 2015;47:1304–15. 26437031 10.1038/ng.3415

[152] Colalillo B, Sali S, Aldouhki AH, Aubry I, Di Marco S, Tremblay ML, et al. An HuR mutant, HuR-V225I, identified in adult T-cell Leukemia/Lymphoma, alters the pro-apoptotic function of HuR. Cell Death Discov. 2024;10:503. 39695179 10.1038/s41420-024-02268-w PMC11655865

[153] Chen Y, McMillan-Ward E, Kong J, Israels SJ, Gibson SB. Oxidative stress induces autophagic cell death independent of apoptosis in transformed and cancer cells. Cell Death Differ. 2008;15:171–82. 17917680 10.1038/sj.cdd.4402233

[154] An X, Yu W, Liu J, Tang D, Yang L, Chen X. Oxidative cell death in cancer: Mechanisms and therapeutic opportunities. Cell Death Dis. 2024;15:556. 39090114 10.1038/s41419-024-06939-5 PMC11294602

[155] Marney CB, Anderson ES, Adnan M, Peng, K-L, Hu Y, Weinhold N, et al. p53-intact cancers escape tumor suppression through loss of long noncoding RNA Dino. Cell Rep. 2021;35:109329. 10.1016/j.celrep.2021.109329 PMC8287872 34192538

[156] Shi XB, Xue L, Yang J, Ma AH, Zhao J, Xu M, et al. An androgen-regulated miRNA suppresses Bak1 expression and induces androgen-independent growth of prostate cancer cells. Proc Natl Acad Sci. 2007;104:19983–8. 18056640 10.1073/pnas.0706641104 PMC2148409

[157] Le MT, Teh C, Shyh-Chang N, Xie H, Zhou B, Korzh V, et al. MicroRNA-125b is a novel negative regulator of p53. Genes Dev. 2009;23:862–76. 19293287 10.1101/gad.1767609 PMC2666337

[158] Nakanishi H, Taccioli C, Palatini J, Fernandez-Cymering C, Cui R, Kim T, et al. Loss of miR-125b-1 contributes to head and neck cancer development by dysregulating TACSTD2 and MAPK pathway. Oncogene. 2014;33:702–12. 23416980 10.1038/onc.2013.13 PMC4294274

[159] Schmitt AM, Garcia JT, Hung T, Flynn RA, Shen Y, Qu K, et al. An inducible long noncoding RNA amplifies DNA damage signaling. Nat Genet. 2016;48:1370–6. 27668660 10.1038/ng.3673 PMC5083181

[160] Kannan K, Munirajan AK, Krishnamurthy J, Bhuvarahamurthy V, Mohanprasad BK, Panishankar KH, et al. The p16INK4alpha/p19ARF gene mutations are infrequent and are mutually exclusive to p53 mutations in Indian oral squamous cell carcinomas. Int J Oncol. 2000;16:585–675. 10675493 10.3892/ijo.16.3.585

[161] Druškovič M, Šuput D, Milisav I. Overexpression of caspase-9 triggers its activation and apoptosis in vitro. Croatian Med J. 2006;47:832–0. PMC2080483 17167855

[162] Muz B, de la Puente P, Azab F, Azab AK. The role of hypoxia in cancer progression, angiogenesis, metastasis, and resistance to therapy. Hypoxia. 2015;3:83–92. 10.2147/HP.S93413 PMC5045092 27774485

[163] Levidou G, Kotta-Loizou I, Tasoulas J, Papadopoulos T, Theocharis S. Clinical significance and biological role of HuR in head and neck carcinomas. Dis Markers. 2018;2018:4020937. 29619127 10.1155/2018/4020937 PMC5829322

[164] Hou H, Sun D, Zhang X. The role of MDM2 amplification and overexpression in therapeutic resistance of malignant tumors. Cancer Cell Int. 2019;19:216. 31440117 10.1186/s12935-019-0937-4 PMC6704499

[165] Essmann F, Engels IH, Totzke G, Schulze-Osthoff K, Jänicke RU. Apoptosis resistance of MCF-7 breast carcinoma cells to ionizing radiation is independent of p53 and cell cycle control but caused by the lack of caspase-3 and a caffeine-inhibitable event. Cancer Res. 2004;64:7065–72. 15466201 10.1158/0008-5472.CAN-04-1082

[166] Zhou R, Frum R, Deb S, Deb SP. The growth arrest function of the human oncoprotein mouse double minute-2 is disabled by downstream mutation in cancer cells. Cancer Res. 2005;65:1839–48. 15753382 10.1158/0008-5472.CAN-03-3755

[167] Portman N, Milioli HH, Alexandrou S, Coulson R, Yong A, Fernandez KJ, et al. MDM2 inhibition in combination with endocrine therapy and CDK4/6 inhibition for the treatment of ER-positive breast cancer. Breast Cancer Res. 2020;22:87. 32787886 10.1186/s13058-020-01318-2 PMC7425060

[168] Joseph D. Targeting novel HuR related apoptotic pathways to prevent cancer progression in cancers with intact p53 genes. Patent application US 63/877,141. 2025.

[169] Joseph D. A novel drug to target an apoptotic pathway for killing cancer cells found in most cancer patients. Patent application US 63/877,144. 2025.

[170] Sato Y, Sugiyama Y, Ishida T, Inufusa H, You F, Joseph D, et al. The Potential Role of Oxidative Stress in Modulating Airway Defensive Reflexes. Antioxidants. 2025;14:568. 40427451 10.3390/antiox14050568 PMC12108395

[171] Joseph D. The Fundamental Neurobiological Mechanism of Oxidative Stress-Related 4E-BP2 Protein Deamidation. Int J Mol Sci. 2024;25:12268. 39596333 10.3390/ijms252212268 PMC11594350

[172] Joseph D. The Unified Theory of Neurodegeneration Pathogenesis Based on Axon Deamidation. Int J Mol Sci. 2025;26:4143. 40362380 10.3390/ijms26094143 PMC12071446

